# Metabolic syndrome: risk factors and molecular drug targets

**DOI:** 10.17179/excli2025-8703

**Published:** 2025-08-29

**Authors:** Rishabh Chalotra, Aniket Gupta, Thakur Gurjeet Singh, Randhir Singh

**Affiliations:** 1 Laboratory of Neuroendocrinology and Metabolic Disorders, Department of Pharmacology, Central University of Punjab, Bathinda, India; 2 Centre for Research Impact & Outcome, Chitkara College of Pharmacy, Chitkara University, Rajpura, 140401, Punjab, India

**Keywords:** metabolic syndrome, molecular targets, asprosin, gut microbiota, AMPK, insulin resistance, visceral adiposity

## Abstract

Metabolic syndrome (MetS), is a non-communicable disorder caused by impaired management and storage of energy, primarily associated with unhealthy diets, sedentary lifestyles and stress. It is diagnosed when any three of the following conditions are observed, obesity (primary factor), hyperglycemia, low HDL, hypertriglyceridemia, and hypertension (ATP III guidelines). MetS affects approximately 14-34 % of the global population, highlighting significant public health concern. If left untreated, it leads to the development of other serious metabolic diseases like atherosclerosis, diabetes, PCOS, NAFLD, NASH, thyroid, cancer, sleep disturbance, osteoarthritis, anxiety, and depression. Despite ongoing research, no first-line drug currently exists for the comprehensive management of MetS. Its multifactorial nature often requires lifelong polytherapy with lifestyle intervention, raising concern over chronic drug use, drug-drug interactions, increasing morbidity and mortality. Therefore, there is a need highlighting the requirement of a single and targeted pharmacotherapy which offers a safer and more specific therapeutic approach. This review aims to identify and analyse ten key molecular targets in managing the pathogenesis of Metabolic Syndrome (MetS). These targets can further pave the way for a targeted and safer approach in the treatment of MetS.

See also the graphical abstract(Fig. 1).

## Key Points

- Metabolic Syndrome is a highly prevalent disorder worldwide, yet no approved, comprehensive treatment currently exists that simultaneously targets all its components.
- Since most of the conditions associated with MetS are chronic, and require lifelong therapy, the risk of drug interaction is elevated, highlighting the need for a single, safer and more effective therapeutic approach.
- Ten molecular targets have been identified and explored for their pharmacological roles, offering the potential to be used individually or in combination for the comprehensive management of MetS. Moreover, several other targets that require further research to clarify their role in MetS have been identified.
- MetS increase the chance of developing ischemic heart disease by 2-3 times and type II diabetes by 5 times. It also promotes the development of several other diseases like atherosclerosis, diabetes, PCOS, NAFLD, cancer, and more.

## Introduction

Metabolic syndrome (MetS), also known as syndrome X is a disorder for storing and managing energy in the body. This non-communicable disorder is characterized by unhealthy diets, sedentary lifestyles, and stress (Fahed et al., 2022[51]). It consists of a cluster of diseases and is diagnosed when an individual exhibits at least three conditions, including obesity (ATP III), a key factor characterized by an increased waist circumference in men (≥ 102 cm/40") and women (≥ 88 cm/35"), hyperglycemia (fasting blood glucose ≥ 100 mg/dL) (Bansal, 2015[8]), Low HDL cholesterol levels in men (≤ 40 mg/dL), and women (≤ 50 mg/dL) (Lee and Siddiqui, 2019[97]), hypertriglyceridemia (fasting triglycerides ≥ 150 mg/dL), and hypertension (≥ 140/90 mm/Hg) (Reaven, 2002[145]; Fahed et al., 2022[51]). Individuals diagnosed with MetS face elevated high risk of developing various metabolic diseases like atherosclerosis, polycystic ovary syndrome (PCOS), osteoarthritis, non-alcoholic steatohepatitis (NASH), non-alcoholic fatty liver disease (NAFLD), diabetes, sleep disturbance, certain types of cancer (colorectal cancer, prostate cancer, breast cancer) (Pothiwala et al., 2009[141]), thyroid imbalance, anxiety, and depression. According to Bhalwar (2020[14]), individuals diagnosed with Metabolic Syndrome (MetS) face 2-3 times high risk of developing ischemic heart diseases and are 5 times more likely to develop type II diabetes than those without MetS (Bhalwar, 2020[14]). 

MetS is one of the most prevalent disorders globally, with an estimated prevalence ranging between 14 % to 34 % (Moore et al., 2017[115]; Fahed et al., 2022[51]). Factors like sedentary lifestyle, modernization, reduced physical activity, dietary habits, and taking stress are responsible for rising incidence of MetS and affecting population in both developed as well as developing countries (Misra and Khurana, 2008[113]). Due to the complexity and multifaced nature of this disorder, the precise pathophysiology of MetS remains unclear. Multiple factors are responsible for the development of MetS and affect various parts of the body. 

Despite ongoing research and development efforts, no established first-line therapeutic agent is found to be effective in the treatment of MetS. Current treatments primarily address individual diseases that make up the syndrome. Many of these conditions are chronic, necessitating lifelong therapy. Although physical exercise is recommended in managing MetS, it is observed that regular physical exercise may not be feasible for all individuals. The prolonged administration of multiple drugs increases the likelihood of drug-drug interactions, potentially leading to additional complications (Suriyapakorn et al., 2019[167]). A study conducted by Khan et al. described the prescription patterns for patients with MetS, where 33.8% of these patients were prescribed three or less, 60.56% patients were prescribed four to six, and 5.63 % patients were prescribed with more than 6 drugs. Considering the severity of drug-drug interactions 17.96 % patients experienced minor interactions, 78.12 % experienced moderate interactions, and around 3.9 % patients experienced major drug interactions (Khan et al., 2020[85]). 

The prescription of multiple drugs may lead to drug-drug interactions, imposing an increased economic burden on patients. Therefore, there is a critical need for a single drug or targeted therapy, to effectively manage this syndrome. Given the complexity of MetS and the need for safer, and more effective therapeutic approaches. This review will further explore the current understanding of MetS pathophysiology, with elucidating potential molecular targets for future therapeutic interventions. 

### Etiology 

**Figure 2:**
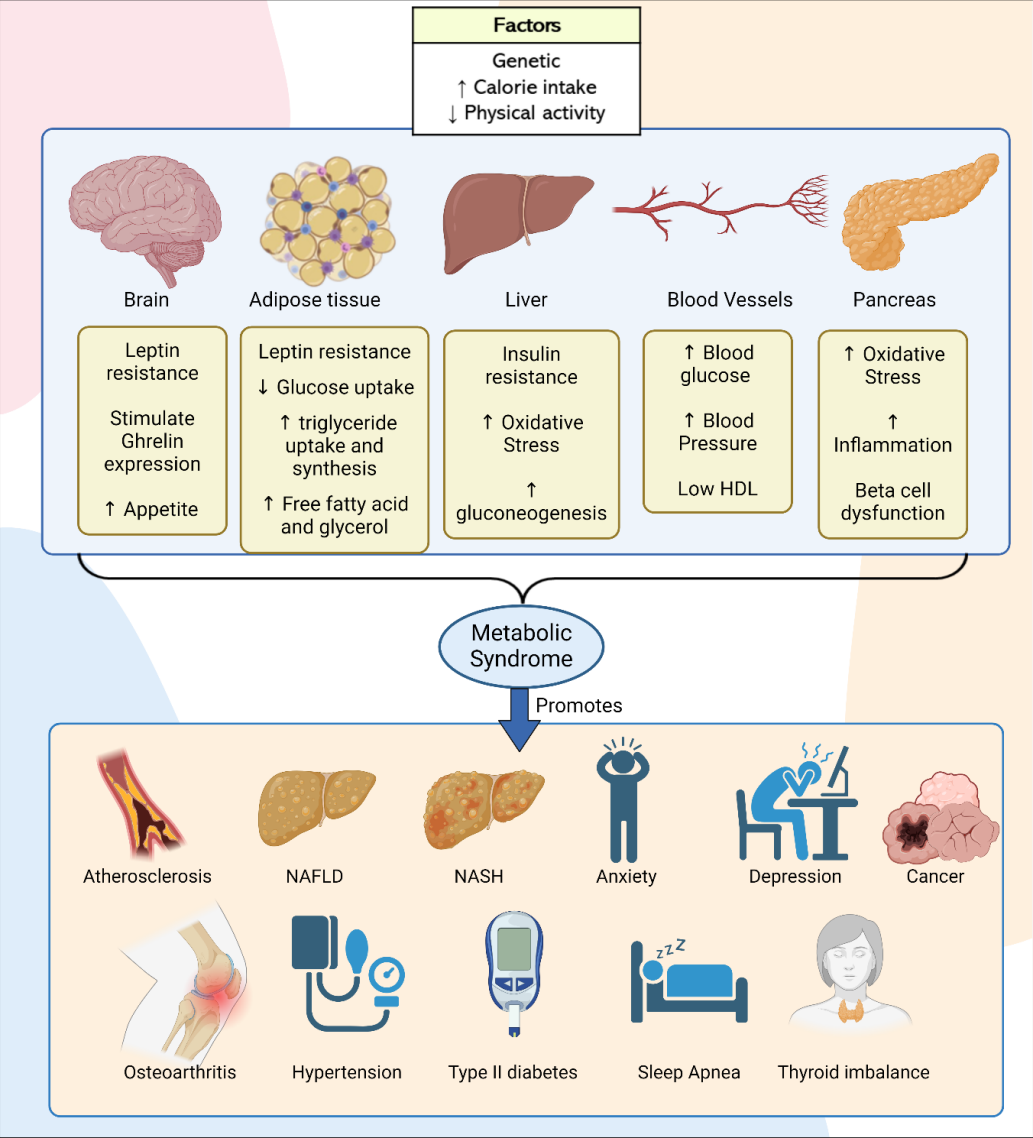
Systemic alterations and disease outcomes linked to MetS. This figure illustrates the key factors contributing to MetS, including genetic, sedentary, and high calorie. It also shows various pathological changes in adipose tissue, liver, pancreas, Blood vessels and brain that lead to MetS. Additionally, it highlights the associated diseases like cardiovascular disease, type II DM, NAFLD, PCOS, and anxiety, emphasizing the interconnected nature of these conditions.

Due to multifaceted nature and association of multiple diseases in the development of MetS, precise pathophysiology of MetS remains unclear. Contributing factors like genetic, improper calorie intake management, reduced physical activity, dysbiosis in gut health, and increased stress, induce pathological changes in various body systems. Key affected systems include the brain, liver, pancreas, adipose tissue, and blood vessels, leading to imbalance in physiological conditions that contribute to the development of MetS. With the development of MetS, the likelihood of developing co-morbidity diseases also increases like type II diabetes, cardiovascular diseases, NAFLD, PCOS, OSA (obstructive sleep apnea), chronic liver disease, osteoarthritis, certain cancers (colorectal, prostate, breast), pregnancy complications, dementia and cognitive impairment, stress, anxiety, oxidative stress, inflammatory mediators (Rochlani et al., 2017[148]; Mendrick et al., 2018[110]; Silveira Rossi et al., 2022[161]) (Figure 2(Fig. 2)).

## Risk Factors for MetS

### Hyperglycemia

It refers to increased fasting blood glucose (≥ 100 mg/dL). When cells are exposed to elevated levels of glucose, there is a reduction in transport of glucose into the cytoplasm to maintain internal glucose homeostasis. However, some cells couldn't maintain rate of glucose transport into cytoplasm, causing intracellular hyperglycemia. This condition results in glucose-induced vascular inflammation (Dandona et al., 2004[37]). Hyperglycemia also impairs, immune system by stimulating the release of cell adhesion molecules and various inflammatory cytokines, further inhibiting leukocyte function (Furnary and Wu, 2006[58]). Additionally, hyperglycemia promotes the production of superoxide in endothelial cells, leading to microvascular damage, endothelial dysfunction and vascular inflammation (Du et al., 2006[46]). Hyperglycemia, indicates a persistent condition of dysregulated glucose levels in bloodstream, which may arise from dysfunctional or compromized beta cell activity, along with peripheral and hepatic insulin resistance. These factors lead to irregular glucose production in the liver. Elevated glucose levels stimulate increased insulin production, resulting in hyperinsulinemia, further exacerbating insulin resistance (Bansal, 2015[8]). Hyperinsulinemia also leads to inadequate lipolysis, causing insufficient fat breakdown and energy consumption, which in turn leads to overeating and fat accumulation. Collectively, it worsens insulin resistance and promotes inflammation, which accelerates the onset and worsening of metabolic imbalances linked to metabolic syndrome (Porte, 1999[140]; Duggal, 2022[47]; Singh et al., 2023[162]). 

### Dyslipidemia 

In MetS, there is an imbalance in the lipid profile, characterized by decreasing fasting HDL levels (Lee and Siddiqui, 2019[97]) and increasing triglyceride levels. A meta-analysis has demonstrated that triglyceride levels function as an independent risk factor for cardiovascular heart disease, even when other factors were also considered (Eckel et al., 2005[50]; Koo et al., 2021[87]). Elevated triglycerides and free fatty acids leads to hyperinsulinemia, which in turn causes insulin resistance (Bays et al., 2005[10]). Insulin plays a significant role in inhibiting lipolysis, breakdown of fats in adipose tissue. However, as insulin resistance increases, hyperglycemia further elevates circulating insulin levels. When adipose tissue becomes resistant to insulin effect, the inhibitory mechanism on lipolysis diminishes, which leads to increased levels of FFA, from adipocytes via hormone sensitive lipase action and through lipoprotein lipase activity in other tissues. The liver then takes up the increased FFA, resulting in increasing the levels of very low-density lipoproteins (VLDL) and increases availability of triglycerides, which contribute in the production of cholesterol. Hypertriglyceridemia also cause change in other lipoproteins, specifically HDL and LDL. All these alterations lead to the development of atherogenic dyslipidemia, a key feature of MetS (Eckel et al., 1995[49]; Menuet et al., 2005[111]). 

### Visceral obesity 

Clinical research on obesity indicates that the pattern of fat distribution in the body, rather than the total amount of fat, is a critical factor for obesity-related health risks. Particularly the accumulation of visceral fat around vital organs like liver, pancreas, and intestine within the abdominal cavity (Di Chiara et al., 2012[45]). Males with a waist circumference of 102 cm (40 inches) or greater and females with 88 cm (35 inches) or greater are classified as having high visceral fat levels, which comprise approximately 10-20 % of the body's total fat (Wajchenberg, 2000[181]). A study using computed tomography to assess adipose tissue, concluded that the accretion of visceral fat significantly contributes to conditions like hyperlipidemia, hypertension, diabetes, and atherosclerosis (Matsuzawa et al., 2004[108]). Another study indicates patients with visceral adiposity despite their mild obesity experience an increased prevalence of coronary artery disease (Nakamura et al., 1994[120]). With an increase in visceral fat, there is a high chance of hypoadiponectinemia and altered adipokine release (Scherer et al., 1995[156]). Hypoadiponectinemia and adipokines could be the major regulators for vascular changes and metabolic disorders, including insulin resistance leading to MetS (Després and Lemieux, 2006[43]; Neeland et al., 2019[123]). 

### Sleep apnea 

OSA is a significant and potentially fatal disorder characterised by repeated disturbances or interruptions in breathing during sleep. It is closely linked to obesity. Excessive body weight contributes to partial or complete collapse in the upper airways, leading to decreased oxygen saturation (Sankri-Tarbichi, 2012[152]). OSA causes repetitive apneas and hypopneas, which results in hypoxia, sleep arousal and hemodynamic changes (Pinto et al., 1993[139]). These disruptions further exacerbate respiratory distress, increasing fatigue, lethargy and cause additional respiratory problems while slowing metabolism. Despite respiratory events, OSA stimulated the stimate nervous system, leading to an increase in both blood pressure and heart rate. The National Commission on Sleep Disorders Research, estimates that sleep apnea contributes to approximately 38,000 cardiovascular fatalities each year (Chokroverty, 2010[30]). Furthermore, OSA raises the 140 % likelihood of heart failure, 60 % of stroke, and 30 % of coronary heart disease (Shahar et al., 2001[160]). Hypertension affects 40 % individuals diagnosed with sleep apnea (Fletcher, 1995[54]). Clinical data from the Mayo clinic indicates that 60 % of patients with sleep apnea also have MetS, compared to 40 % of those without the condition (Parish et al., 2007[133]). Patients with sleep apnea experience repetitive hemodynamic oscillations during night, impacting blood pressure, heart rate, and cardiac function (Nieto et al., 2000[127]). Hypoventilation is often observed in these patients, which contributes to obesity, excessive daytime sleepiness, and the activation of systemic arterial hypertension. All these factors leads in the progression of MetS (Gami et al., 2003[60]). 

### Hypertension

Elevated blood pressure is a critical and prevalent factor for the diagnosis of MetS, with approximately 80 % of MetS patients experiencing hypertension (Chimonas et al., 2010[28]). The co-existence of MetS and hypertension significantly enhances the risk of developing end-organ damage, including intima-media thickness, microalbuminuria, hypertrophy, and hypertensive retinopathy (Athyros and Mikhailidis, 2015[4]; Katsimardou et al., 2020[81]). Various pathological mechanisms have been suggested for the onset of MetS. These mechanisms include insulin resistance, stimulation of sympathetic NS, reduced Endothelial Nitric oxide synthase activity, dysregulation of adipokines, obesity and sodium retention (Kotsis et al., 2018[90]). Insulin resistance is a major factor in activating the SNS by increasing expression of angiotensin II receptors and reducing nitric oxide (NO) synthesis, which contributes to elevated heart rate and blood pressure (Mancia et al., 2007[106]; Tziomalos et al., 2010[174]; Chauhan et al., 2023[27]). Furthermore, excessive leptin secretion stimulates the hypothalamic-pituitary-adrenal axis, combined with obstructive sleep apnea and baroreflex dysfunction, further activates the sympathetic nervous system (Mancia et al., 2007[106], Schlaich et al., 2015[157]). Dysregulation of adipokines, which are signalling molecules released by adipocytes, and promotes inflammation and worsening insulin resistance. Obese patients exhibited an increase in renal tubular reabsorption, leading to retention of sodium, which further increases the risk in development of MetS (Kotsis et al., 2010[89]). 

### Metabolic acidosis

Metabolic acidosis is a disorder characterised by an imbalance in the body's acid-base homeostasis, resulting in a decrease in blood pH due to a reduction of serum bicarbonate concentration. This condition arises from the accumulation of acids (anions) or the loss of bicarbonate from the kidneys. To maintain normal pH, kidneys reabsorb all filtered bicarbonate (HCO_3_^-^) in the proximal tubule and excrete daily H^+ ^ions from the collecting duct. The proximal tubule absorbs approximately 80 % of the filtered HCO_3_^-^, 10 % is absorbed by the ascending loop of Henle, and the remaining is absorbed by distil convoluted tubule (DCT) (Rector, 1983[146]). Daily acid excretion (50-80 mEq of H+) occurs through free H+ excretion, ammonium excretion, or titratable acidity. The urine pH can decrease as low as 5, indicating maximum acidification. A urine volume of 3 liters will contain approximately 0.03 mEq of free H+, highlighting inadequate H+ excretion. Therefore, renal mechanisms involved in acid-base balance, including kidney acid balance and ammonium production, play a significant role (Verlander et al., 1988[178]). Severe acidosis can lead to cardiovascular complications like hypotension due to decreased peripheral vascular resistance, reduced cardiac output and an increased predisposition to cardiac arrhythmias. Additionally, it can result in reduced hepatic blood flow, insulin resistance, elevated calcium levels, decreased ATP synthesis, and gastric atony, where the stomach loses its muscle tone, leading to digestive issues (Kraut and Madias, 2010[92]; Celotto et al., 2016[19]).

### Insulin resistance 

Insulin resistance is a metabolic condition characterised by reduced cellular responsiveness to the insulin hormone, which is secreted from pancreas and has role in blood glucose homeostasis. To compensate for elevated glucose levels, pancreatic beta cells secrete more insulin (hyperinsulinemia) to maintain euglycemia. However, with time, beta cells may be unable to cooperate with increased insulin production. As a result, insulin levels become insufficient to overcome this resistance, leading to hyperglycemia. This failed compensatory mechanism, when coupled with IR, leads to the development of type II DM (Wilcox, 2005[183]; Chalotra et al., 2024[23]). Considering its major targets, IR in skeletal muscles impairs insulin signalling pathways, reducing GLUT4 translocation to cell membrane, thereby decreasing glucose uptake. In the liver, it may cause an increase in hepatic glucose levels. Normally, FFA which are derived through triglycerides breakdown in adipose tissue, by the influence of cAMP, which is elevated during lipolysis, particularly during fasting conditions (Roberts et al., 2013[147]). Postprandially, insulin typically inhibits lipolysis by reducing cAMP activity (Alberti et al., 2005[1]; Savage et al., 2007[154]). Whereas in a state of insulin resistance, this inhibitory effect is diminished, causing increased lipolysis and FFA levels. Increased FFA leads to hepatic insulin resistance by promoting the production of glucose, triglycerides, apolipoprotein B (apoB), and VLDL, which are atherogenic and contribute to cardiovascular diseases (Zhao et al., 2020[190]). In adipose tissue, insulin resistance leads to dysregulated lipolysis, causing the release of excessive FFA, contributing to lipotoxic effects on other tissues, and exacerbating metabolic syndrome (Jensen et al., 1989[79]; Choi et al., 2010[29]). Insulin resistance also causes an increase in mild inflammation characterised by increased levels of inflammatory cytokines and oxidative stress. It also promotes alteration in mitochondrial functions, reduced oxidative capacity, and increased lipid accumulation. All these conditions further leads in the development of MetS (Dandona et al., 2004[37]; Hurrle and Hsu, 2017[74]; Chalotra et al., 2024[22]). 

## Molecular Targets of MetS

Several molecular targets have been identified for their roles in addressing metabolic diseases that collectively contribute to the development of MetS. These targets are key regulators of obesity, dyslipidemia, hypertension, and hyperglycemia conditions that define MetS. According to the criteria for MetS, the diagnosis requires the presence of at least three factors, one of which is obesity. In this study, only those molecular targets that have a direct or indirect role in influencing at least three of these conditions, with obesity as a key factor, are considered. These targets include:

### AMPK

Adenosine 5' monophosphate activated protein kinase (AMPK) is a critical cellular energy sensor in cells which maintains energy homeostasis. AMPK is activated when the AMP/ATP ratio increases, which occurs during conditions like hypoxia, metabolic stress, and glucose deprivation. AMPK has a significant contribution in the metabolism of lipids and glucose, cell survival, growth and inflammation. AMPK activation promotes catabolic pathways which produce ATP with inhibition of anabolic processes which requires ATP. Activation of AMPK is mediated via two primary signals, AMP-dependent pathway via LKB1 and another by Ca2+ dependent mediated by CaMKKβ (Sanders et al., 2007[151]). AMPK serves as a central regulator of sensing cellular energy levels and becoming activated through mechanisms involved in allosteric and phosphorylation regulation by AMP. In the liver, AMPK modulates glucose balance by downregulating gluconeogenesis, both at the gene expression level and by reducing hepatic glucose production. AMPK activation enhances the phosphorylation of GSK-3β, while concurrently decreasing cAMP response element transcriptional activity and supressing the expression of PEPCK-C, thus inhibiting gluconeogenesis (Horike et al., 2008[72]). 

Chronic AMPK activation has increased the translocation of Glut4 in plasma membrane, increasing the uptake of glucose, and glycogen content in skeletal muscles, and adipose tissue (Holmes et al., 1999[71]). AMPK activates and phosphorylates eNOS, promoting production of NO, which is a vasodilator that maintains homeostasis in blood vessels and regulates blood pressure (Greig et al., 2015[64]). AMPK also inhibits the HMG-CoA reductase, in biosynthesis of cholesterol, thereby lowering LDL levels (Loh et al., 2019[103]). Additionally, AMPK inhibits acetyl-CoA carboxylase, leading to a reduction in malonyl-CoA levels, which subsequently relieves the inhibition of carnitine palmitoyltransferase 1 (CPT-1), an essential enzyme involved in fatty acid oxidation (Pimenta et al., 2008[138]). AMPK also reduces mitochondrial ROS generation while mitochondrial genes in skeletal muscles, increasing NAD+ and sirtuin 1, leading to the activation and deacetylation of PGC-1α. Muscle mitochondrial dysfunction is also linked to both IR and metabolic inflexibility in type II DM and obesity, indicating that AMPK-driven mitochondrial enhancement may help alleviate these conditions (Narkar et al., 2008[121]). AMPK also regulates PAI-1 which has role in adipose tissue dysfunction, causing inflammation and insulin resistance; it is a crucial biomarker for MetS (Nawaz and Siddiqui, 2022[122]). 

**Figure 3:**
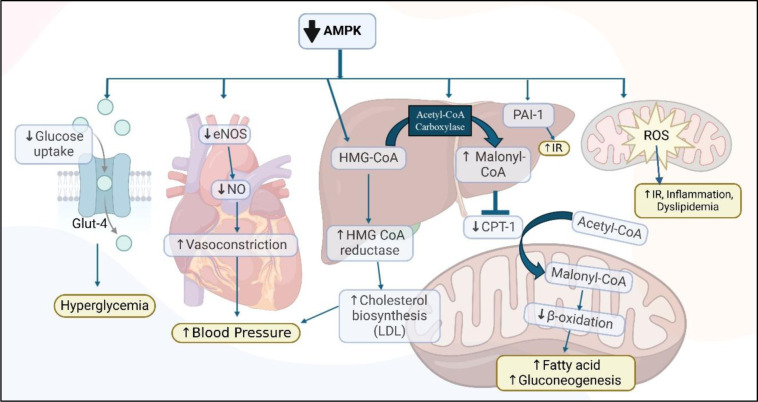
Involvement of AMPK in the pathogenesis of MetS: AMPK: AMP-Activated Protein Kinase; HMG CoA: 3-Hydroxy-3-Methylglutaryl Coenzyme A; eNOS: Endothelial Nitric Oxide Synthase; Glut4: Glucose Transporter Type 4; LDL: Low-Density Lipoprotein; CPT: Carnitine Palmitoyltransferase; Acyl CoA: Acyl Coenzyme A; ROS: Reactive Oxygen Species; NO: Nitric Oxide;

In MetS, there is dysregulation of AMPK activity, which significantly contributes to the pathogenesis of MetS through multiple mechanisms, including impaired glucose uptake, increased blood pressure, dyslipidemia, increased oxidative stress, and decreased fatty acid oxidation. Several pharmacological agents and hormones have been reported to activate AMPK in vivo. Changes to mitochondrial efficiency and cellular energy levels may contribute to energy imbalances and play a role in the activation of AMPK (Zhang et al., 2009[189]) (Figure 3(Fig. 3)).

**Role in MetS: **AMPK integrates the regulatory effects of obesity, dyslipidemia, hypertension, and hyperglycemia which collectively manages MetS. AMPK mitigates obesity by enhancing fatty acid oxidation and mitochondrial function via inhibition of acetyl-CoA carboxylase and promotion of carnitine palmitoyltransferase 1 activity (Pimenta et al., 2008[138]). It manages dyslipidemia by reducing LDL cholesterol levels via inhibition of HMG-CoA reductase and decreasing triglyceride levels through suppression of lipid biosynthesis (Loh et al., 2019[103]). Hypertension is managed through activation of eNOS, causing NO production with improved vascular function (Greig et al., 2015[64]). Hyperglycemia by inhibiting hepatic gluconeogenesis and enhancing glucose uptake in peripheral tissues via increased Glut4 translocation (Holmes et al., 1999[71]). Insulin Resistance by lowering the levels of PAI-1 and ROS (Nawaz and Siddiqui, 2022[122]). This comprehensive regulation of energy metabolism, lipid profiles, vascular health, and glucose homeostasis highlights AMPK's role in mitigating the complex pathophysiology of MetS. 

### GLP-1:

Glucagon-like peptide-1 is a multifaceted hormone secreted by L-cells of intestines in response to glucose and various nutrients. It is predominantly expressed in lungs, blood vessels, heart, breast, kidney, pancreas, CNS and GIT (Körner et al., 2007[88]). In pancreatic β-cells, GLP-1 regulates insulin expression in response to elevated glucose levels, which is its primary function. Besides its wide-ranging pharmacological roles, GLP-1 has numerous metabolic effects. The GLP-1 receptor is involved in slow gastric emptying, glucagon secretion, increased satiety, and promoting pancreatic β-cell mediated insulin secretion. This receptor primarily modulates elevated blood glucose levels, playing a crucial role in maintaining glucose homeostasis (DeFronzo, 2009[41]; Cryer, 2011[35]; Pettersson et al., 2011[136]). 

GLP-1 receptor agonists, commonly used for the treatment of obesity and type-II DM, often referred to as incretin mimetics (Collins and Costello, 2019[34]). Both glucose-dependent insulinotropic polypeptide (GIP) and GLP-1 incretin hormones, are degraded by DPP-4. Both GIP and GLP-1 promote insulin secretion in response to oral intake of glucose, a phenomenon known as the incretin effect (Davidson, 2011[39]; Vilsbøll et al., 2012[180]). However, in type-II diabetes, this effect diminishes, though GLP-1 can revives insulin secretion, making it a valuable therapy for this condition. In managing type-II diabetes, GLP-1 RA offers multiple benefits like delayed gastric emptying that regulates the absorption of nutrients; inhibition of glucagon production from pancreatic α-cells that helps in controlling blood sugar levels. Additionally, they preserve and promote pancreatic β-cell, stimulating their proliferation. Patients using GLP-1 RAs often experience weight loss and a lowering of BP (Gallwitz, 2011[59]; Garber, 2011[61]; Okerson and Chilton, 2012[131]). Furthermore, GLP-1 RAs improve various cardiac functions, including myocardial contractility, endothelial function, coronary blood flow, left ventricular ejection fraction, cardiac output, while minimizing infarction size (Mannucci and Monami, 2017[107]; Zheng et al., 2018[191]). In kidneys GLP-1 increase in sodium excretion (Gutzwiller et al., 2006[68]). It also increases the glucose uptake in muscles and decrease glucose production in the liver. GLP-1 RA also regulates neuroprotective effects and increased satiety via the hypothalamus. Overall, these medications offer a decrease in mortality rate, leading to a comprehensive treatment option for type II diabetes and obesity, which are two major factors in MetS (Seufert and Gallwitz, 2014[159]; Hinnen, 2017[70]). 

**Role in MetS: **GLP-1 receptor agonists (RAs) are crucial in managing MetS. These agents boost insulin secretion in response to glucose levels, improving glycemic control and managing hyperglycemia (Davidson, 2011[39]; Vilsbøll et al, 2012[180]). Additionally, GLP-1 RAs regulate and lowers gastric emptying time and promote satiety, leading to reduced food intake and subsequently weight loss, which directly combats obesity. Though the suppression of glucagon release from pancreatic α-cells, GLP-1 RAs aid in stabilizing blood glucose levels and lowers risk of dyslipidemia (Gallwitz, 2011[59]; Garber, 2011[61]; Okerson and Chilton, 2012[131]). Furthermore, they have positive cardiovascular effects by reducing BP, enhancing endothelial function, and increasing cardiac output (Mannucci and Monami, 2017[107]; Zheng et al., 2018[191]). The multifaceted actions of GLP-1 RAs, including their influence on renal sodium excretion and liver glucose production, contribute to their efficacy in managing MetS. 

### SGLT-2: 

The kidneys play a crucial role in glucose metabolism; filtering roughly 180 grams of glucose daily through glomeruli, with nearly all of which is resorbed in the renal proximal convoluted tubule (RPCT). This reabsorption accounts for 90-95 % of filtered glucose is primarily facilitated by sodium-glucose co-transporter 2 (SGLT2), located in the S1 segment of the proximal tubular epithelial cells in kidneys (Chao and Henry, 2010[25]). SGLT-2 is also expressed in the alpha cells of pancreatic islets. SGLT2 inhibitors are used in managing type II DM due to their multiple beneficial effects, including glucosuria and osmotic diuresis, which result in increased urinary glucose excretion and diuresis, respectively. This mechanism helps lower blood glucose levels, reduces body fat, and improves metabolic profile. Additionally, SGLT2 inhibitors contribute to lowering high blood pressure and have renal and cardiac protective properties (Premji et al., 2022[142]). 

However, SGLT2 inhibitors are associated with potential adverse events, such as volume depletion and euglycemic diabetic ketoacidosis. This condition appears to be related to elevated glucagon levels, which promote the β-oxidation of fatty acids and hepatic ketone production. Other risks include genital infections, urosepsis, and amputations associated with certain drugs in this class. Overall, it's important to note that extent of weight loss is modest; in initial four weeks of treatment, water and electrolytes may be lost, followed by preferential loss of fat mass (Gharaibeh et al., 2019[62]). Despite these risks, SGLT2 inhibitors have positively affected various health parameters, including reductions in total and visceral fat mass, body weight, and serum ALT and AST levels. Even with increased food intake, increased urinary calorie loss offsets this effect. Furthermore, these inhibitors lead to decreased glucose and insulin levels in the body. 

**Role in MetS:** SGLT2 inhibitors show a role in managing metabolic syndrome. By inhibiting SGLT2 in the RPCT, these agents enhance glucosuria and osmotic diuresis, leading to increased urinary glucose excretion and subsequent reductions in blood glucose levels and body fat (Premji et al., 2022[142]). This mechanism not only lowers hyperglycemia but also promotes modest weight loss by preferentially reducing fat mass (Ni et al., 2020[126]). SGLT2 inhibitors additionally lower blood pressure through their diuretic effect, contributing to better hypertension management (Premji et al., 2022[142]). Moreover, these drugs improve the metabolic profile by reducing visceral fat, AST and ALT (Premji et al., 2022[142]). Despite potential adverse events such as volume depletion and euglycemic diabetic ketoacidosis, the overall benefits of SGLT2 inhibitors, including their renal and cardiac protective properties, make them valuable for addressing the multifaceted components of metabolic syndrome (Premji et al., 2022[142]).

### HMG-CoA

Hydroxymethylglutaryl coenzyme A, also known as HMG-CoA, is a metabolic intermediate involved in various metabolic pathways, mainly the mevalonate and ketogenic pathways (Bhagavan and Ha, 2011[13]). The mevalonate pathway includes the conversion of acetyl CoA to HMG-CoA, which later converts into mevalonic acid, which ultimately leads to the biosynthesis of cholesterol, steroid hormones, and bile, where excessive accumulation of cholesterol leads to dyslipidemia, increasing cardiovascular risks, and insulin resistance (Pihlajamäki et al., 2004[137]); glucocorticoid hormone like cortisol promote obesity, insulin resistance, and hypertension (Varughese et al., 2014[177]; Beaupere et al., 2021[11]). Bile, an important regulator of systemic metabolism, plays a role in the absorption of fat and modulation of cholesterol and glucose metabolism by FXR and TGR5/M- BAR regulators (González-Regueiro et al., 2018[63]). Drugs like statins used as HMG-CoA reductase inhibitors, block the conversion of HMG-CoA to mevalonic acid. These are utilized for both primary as well as secondary prevention of coronary heart diseases, and are highly effective in managing hypercholesterolemia, by lowering total cholesterol, low-density lipoprotein, and triglycerides, while raising high-density lipoprotein (Stancu and Sima, 2001[164]). The reduction in intercellular cholesterol triggers the activation of sterol regulatory element for binding protein (SREBP), which then migrates to the nucleus, where it binds to sterol response elements, leading to the conversion of cholesterol into bile salts (Mausner-Fainberg et al., 2008[109]; Bansal and Cassagnol, 2019[7]). Besides lowering lipid concentrations, HMG-CoA reductase inhibitors provide cardiovascular protection by maintaining the integrity of atherosclerotic plague, reducing inflammation and CRP levels, improving endothelial functions via eNOS activity and decreasing thrombogenicity by inhibiting platelet activity and thromboxane A2 synthesis (Anderson et al., 1995[3]; Rosenson and Brown, 2002[149]). 

Furthermore HMG-CoA in the presence of HMG-CoA lyase converts to acetoacetate and also releases acetyl CoA. This acetoacetate converts into ketone bodies like acetone and D-β-hydroxybutyrate (Bhagavan and Ha, 2011[13]). Ketone bodies have multiple pharmacological actions in MetS. They act as an alternate fuel for the body. Ketone bodies also promote resistance to inflammatory and oxidative stress, with various cell protective mechanisms like NRF-2, and AMPK. In heart, ketone bodies have shown improvement in resistance to the cardiotoxic effect of doxorubicin (Kolb et al., 2021[86]; Liu et al., 2021[101]). 

**Role in MetS: **HMG-CoA plays important role in MetS through its involvement in mevalonate and ketogenic pathways. In mevalonate pathway, the HMG-CoA is converted to mevalonic acid, leading to cholesterol synthesis (Pihlajamäki et al., 2004[137]). Excess cholesterol could lead to dyslipidemia, obesity, hypertension, hyperglycemia, which are key components of MetS. Additionally, HMG-CoA lyase converts HMG-CoA to acetoacetate, forming ketone bodies like D-β-hydroxybutyrate, which act as alternative fuels, improving insulin sensitivity, reducing inflammatory and oxidative stress, improving cardiovascular protection and aids in weight management (Bhagavan and Ha, 2011[13]; Kolb et al., 2021[86]; Liu et al., 2021[101]). Which all together contributes to manage MetS. 

### Leptin

Leptin is a polypeptide hormone encoded by the ob/lep gene and secreted mainly from white adipose tissue. As an adipocytokine, It plays a vital role in controlling energy expenditure, food intake, and overall metabolism by acting through receptors located on hypothalamus. Leptin primarily maintains energy homeostasis by inhibiting orexigenic neuropeptides such as peptide YY (PYY) and neuropeptide Y (NPY) (Kaye et al., 1998[83]), and activating anorexigenic pathways involving neuropeptides like pro-opiomelanocortin (POMC) and corticotropin-releasing factor (CRF) (Woods and D'Alessio, 2008[184]), which increases energy expenditure and reducing appetite, thereby regulating body weight (Halaas et al., 1995[69]). In CNS, leptin interactions with receptors supports these functions, while in the periphery, leptin modulates insulin sensitivity and glucose uptake highlighting its crucial role in glucose metabolism. 

Beyond the energy regulation, leptin exhibit pleotropic effects, impacting a variety of physiological processes. It influences hematopoiesis and neurogenesis, and neuroprotection by modulating neuronal activity and survival. Leptin also exert immunomodulatory effects by regulating inflammatory responses and maintaining immune balance. Additionally, it plays role in cardiovascular function, regulating blood pressure, glomerular filtration rate, and gastric emptying. Leptin has been found in the gastric mucosa and fundic glands, suggesting a role in gastrointestinal function as well (de Candia et al., 2021[40]). 

Leptin functions through multiple receptor isoforms categorized as *ObR* a, *ObR* b, c, d, e, and f (Lee et al., 1996[95]). The *ob* gene, encodes a 167 amino acid protein, which includes a 21 amino acids signal peptide, This gene displays an 84 % sequence similarity with the mouse *ob* gene (Kaye et al., 2000[84]; Janečková, 2001[78]). Upon binding to its receptor, leptin activates multiple intercellular signalling pathways contributing to its regulatory functions across various organs, including brain, adipose tissue, muscles and kidneys. Additionally, it inhibits water intake and may contribute to long-term increases in blood pressure (Janečková, 2001[78]), further contributing to its cardiovascular effects. A study suggests that low doses of leptin (100 ng/kg to 2 μg/kg) decrease the heart rate, whereas at (> 100 μg/kg), it initially decreases and then increases heart rate, possibly due to stimulation of the sympathetic NS (Lin et al., 2015[100]). 

Leptin dysregulation is critical in pathophysiology of obesity, type II DM and cardiovascular diseases. In obesity and type II diabetes, leptin levels are elevated (Belhayara et al., 2019[12]), reflecting a state of leptin resistance, where its effectiveness in regulating appetite and metabolism is diminished (Vilariño-García et al., 2024[179]). Elevated serum leptin concentrations are linked to increased intima-media thickness in the carotid artery, contributing to atherosclerosis and adverse cardiac remodelling in coronary artery disease (Ciccone et al., 2001[31]). In MetS, leptin resistance causes weight gain, insulin resistance, and other metabolic changes by impairing anorexigenic signalling, reduced satiety, and increased energy storage (Kaye et al., 1998[83], 2000[84]). 

Leptin has role in regulation of thyroid hormone, influencing the hypothalamus hypothalamus-pituitary-thyroid axis. Disruption of leptin signaling can affect the feedbalck mechanism between thyroid hormone T4/T3 and the hypothalamus-pituitary axis, potentially altering metabolic rate (Flier et al., 2000[55]). Moreover, leptin modulates glucose homeostasis by suppressing glucose-stimulated insulin secretion via sympathetic nervous system activation, highlighting its interconnected role in metabolic regulation (Coll and Yeo, 2013[33]). Biguanide hypoglycemic agents has role in reducing insulin resistance whereas second generation sulfonylureas increase leptin levels in obese patients, offering role in managing metabolic imbalance and leptin dysregulation (Paz-Filho ete al., 2012[134]). Leptin is a major regulator of metabolism and energy homeostasis, having effects on the cardiovascular, immune, and neurological systems. Dysregulation of leptin signaling, underscores its importance in pathogenesis of cardiovascular as well as metabolic diseases (Vilariño-García et al., 2024[179]).

**Role in MetS:**

Leptin, a crucial regulator of energy homeostasis by regulating metabolism, food intake, and energy expenditure through hypothalamic receptors. Elevated leptin levels are commonly observed in obesity and TIIDM, which are associated with increased cardiovascular risks such as atherosclerosis and adverse cardiac remodelling (Ciccone et al., 2001[31]; Belhayara et al., 2019[12]). Leptin influences various physiological processes, including glucose metabolism, blood pressure regulation, and neuroendocrine functions, by interacting with multiple leptin receptor isoforms (Ob-R) (Janečková, 2001[78]) (Lee et al., 1996[95]). Leptin also regulates orexigenic and anorexigenic neuropeptides which are important in controlling appetite and energy balance and cause effect on insulin sensitivity and cardiovascular health, highlighting its contribution to the pathophysiology of MetS (Woods and D'Alessio, 2008[184]). The dysregulation of leptin signalling causes impaired energy balance and metabolic disturbances, leading to development and progression of MetS.

### Asprosin 

Asprosin is an adipokine derived from C-terminal segment of profibrillin-1 protein. encoded by exon 65 and exon 66 of the fibrillin-1 gene on the chromosome 15q21.1. The presence of FBN1 mRNA in white adipose tissue indicates that this tissue is a primary site for asprosin production (Yuan et al., 2020[187]). It is thought to play play in regulating adipocyte browning. Recent research has identified the measurable amount of asprosin in several body fluids including saliva, urine, serum, plasma, and breast milk (Ugur and Aydin, 2019[175]; Morcos et al., 2022[116]). 

**Figure 4:**
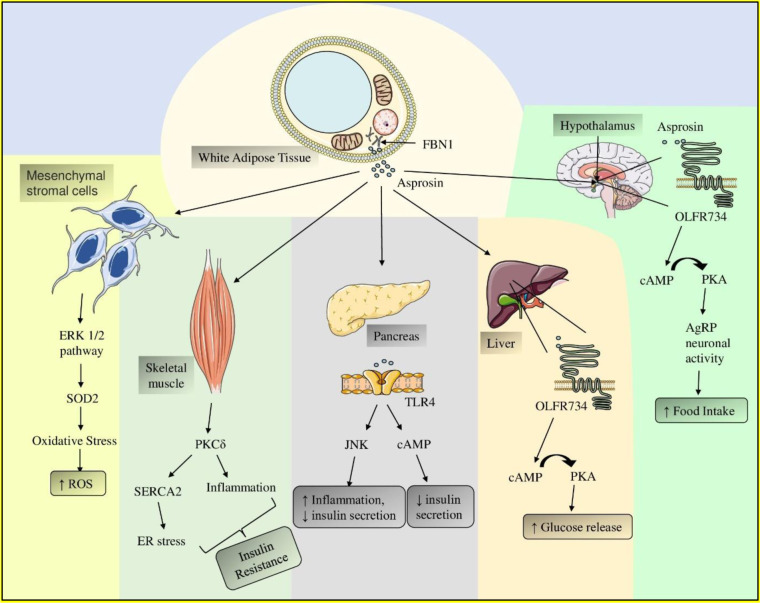
Asprosin-Mediated Mechanisms in Developing MetS

After secretion, asprosin exerts both central modulating as well as peripheral effects. Centrally it functions as an appetite stimulant hormone by acting on olfactory receptor 4M1 (OR4M1) (Farrag et al., 2023[53]). In the pancreas, asprosin interacts with TLR4, which causes activation of two pathways, JNK and cAMP, leading to decreased insulin secretion and increased inflammation. In skeletal muscle, asprosin induces IR via PKCδ/SERCA2 pathway. In Mesenchymal cells, asprosin activates the ERK1/2 pathway, producing reactive oxygen species (ROS) and promoting inflammation (Ugur and Aydin, 2019[175]; Morcos et al., 2022[116]; Figure 4(Fig. 4)). 

**Role in MetS: **Asprosin stimulates appetite via olfactory receptor 4M1, contributing to obesity. In the pancreas, it binds to toll-like receptor 4 (TLR4), activating JNK and cAMP pathways, leading to increased inflammation and decreased insulin secretion, causing hyperglycemia. In skeletal muscle, it induces insulin resistance via the PKCδ/SERCA2 pathway. In mesenchymal cells, it activates the ERK1/2 pathway, producing reactive oxygen species (ROS) and promoting inflammation, exacerbating MetS. All these conditions contribute in the management of MetS (Ugur and Aydin, 2019[175]; Morcos et al., 2022[116]). 

### Phosphate dysregulation

Phosphorus is a highly abundant minerals, crucial for intercellular signaling (such as phosphorylation), energy production in the form of ATP, formation of cellular membranes (phospholipids), and the synthesis of nucleic acids (DNA and RNA) (Razzaque 2009[144], 2011[143]). The dietary recommendation for phosphorus intake in adults in the U.S. is set at 700 mg/day. However, about one-third of the U.S. population exceeds this amount (Chang et al., 2014[24]), which has linked to elevated salivary IL-1β and reduced IL-4 levels, potentially contributing to increased inflammation. 

Fibroblast growth factor 23 (FGF23), an important phosphate homeostasis regulator, produced by bone cells, undergoes post-translational modifications including (1) phosphorylation by FAM20C at serine 180 to induce cleavage and (2) O-glycosylation by GALNT3 on threonine 178 to prevent cleavage (Ichikawa et al., 2009[75]; Tagliabracci et al., 2014[169]). FGF23 interacts with the FGFR/ αKlotho complex in renal cells, which reduces the expression of NaPi-2a, leading to decreased phosphate reabsorption in the kidneys and enhanced phosphate excretion in the urine. It further modulates vitamin D activation, which further influences phosphate and calcium balance in the body. 

Disrupted phosphate regulation elevates the risk of systemic organ dysfunction association with metabolic syndrome. Hyperphosphatemia causes low-grade inflammation, contributing to vascular stiffness, and hypertension. These changes adversely affect glucose regulation, potentially leading to diabetes. Additionally, abnormal metabolism of lipid from high phosphate levels can contribute to obesity, a significant factor in MetS (Lacerda-Abreu and Meyer-Fernandes, 2021[93]). Excessive phosphorus intake raises IL-1β levels, which impairs pancreatic β-cell function by inducing fasting-induced apoptosis via NF-κB activation (Maedler et al., 2002[104]). This process disrupts insulin production and secretion, further exacerbating metabolic issues. Its important to recognize that serum phosphate levels do not provide an accurate reflection of total body phosphate, as the intervascular phosphate pool accounts for less than 1% of the total body phosphate content (Mousa et al., 2018[117]). 

Excessive phosphate intake may leads to toxicity, potentially causing chronic inflammation by inducing cytotoxicity effects and interfering with subcellular signalling pathways (Mironov et al., 2022[112]). This can lead to persistent inflammation and damage to cellular structures. A high phosphate diet can alter the gut microbial ecosystem, promoting conditions associated with metabolic syndrome. Various studies demonstrate a linkage in gut microbiota with MetS via inflammatory pathways (Chassaing and Gewirtz, 2014[26]; Ojo et al., 2021[130]). *In-vitro *studies have demonstrated the role of oxidative stress associated with uptake of phosphate in mitochondria, which contributes to impaired insulin synthesis and secretion (Nguyen et al., 2015[125]). Tonelli et al. found a correlation between elevated serum phosphate levels and increased cardiovascular risk (Tonelli et al., 2005[171]). Individuals with serum phosphate level of 3.5 mg/dL were shown to have 1.55 times higher hazard ratios compared to those with levels below 2.8 mg/dL. While serum levels under 4mg/dL are generally considered within the normal range, the Mayo Clinic suggests a reference range of 2.5 - 4.5 mg/dL (Tonelli et al., 2005[171]). 

**Figure 5:**
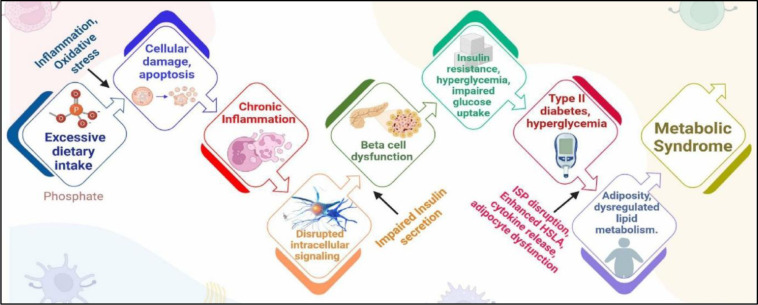
Phosphate dysregulation to MetS: a sequential overview; ISP: Insulin Signalling Pathway; HSLA: Hormone-Sensitive Lipase Activity;

**Role in MetS: **Dysregulated phosphate levels, primarily by excessive dietary intake, induce low-grade inflammation, vascular stiffness, hypertension, while disrupting glucose and lipid metabolism, thereby promoting insulin resistance, diabetes and obesity (Lacerda-Abreu and Meyer-Fernandes, 2021[93]). The FGF23 regulates phosphate levels, highlighting the connection between phosphate homeostasis and metabolic health. FGF23 modulates renal phosphate reabsorption and vitamin D activation, and its imbalance has been linked to a higher risk of cardiovascular diseases (Ni et al., 2020[126]). Additionally, it affects gut microbiota, linking gut health to MetS via inflammatory pathways (Chassaing and Gewirtz, 201[26]; Ojo et al., 2021[130]). Elevated serum phosphate levels correlate with heightened cardiovascular risks, underscoring the importance of maintaining balanced phosphate levels to mitigate MetS-related complications (Figure 5(Fig. 5)).

### Inflammatory and oxidative mediators

MetS is recognised as a proinflammatory and prothrombotic state, where adipose tissue, being a major contributor to its pathophysiology. Adipose tissue serves as both an endocrine and paracrine organ. In response to excessive nutrition, adipocytes undergo hypertrophy and hyperplasia, which can sometimes exceed the capacity of the local blood supply, leading to a hypoxic state. Hypoxia can induce macrophage infiltration, cell necrosis, and production of adipokines, including pro-inflammatory cytokines and markers of inflammation (Sypniewska, 2007[168]; Coelho et al., 2013[32]), which leads to the development of oxidative stress and inflammation (Gupta et al., 2025[67]; Netzer et al., 2015[124]). Adipokines, production like monocyte chemoattractant protein-1, adiponectin, leptin, irisin, TNF-α, and IL-6, are found to be dysregulated in MetS (Savaş et al., 2020[155]). Several inflammatory markers that influence MetS and obesity including proinflammatory cytokines IL-6, TNF-α, and IL-1β, which are primarily produced due to macrophage infiltration triggered by obesity in adipose tissue (Nishimura et al., 2009[128]). Studies suggest that in diabetes and obesity, there is an increase in inflammatory cytokines, and neutralization of these inflammatory cytokines improves insulin action in obese rat models (Lo et al., 2007[102]). Study suggest that the obese mice deficit of TNF-α and iNOS exhibit improved insulin sensitivity compared to control obese animals (Uysal et al., 1997[176]). Since, insulin resistance is linked to elevated inflammatory markers, number of inflammatory markers gets elevated in MetS like the IL-6, leukocyte count, TNF- α and CRP (Schmidt et al., 1999[158]; Duncan et al., 2003[48]). Study indicate that patients with mild hyperglycemia, like impaired glucose tolerance also experience inflammation (Müller et al., 2002[118]). Other diseases like hyperglycemia, hypertension, and even prehypertension are associated with markers of inflammation (Kaplan and Frishman, 2001[80]; Lee and Pratley, 2005[98]). 

**Figure 6:**
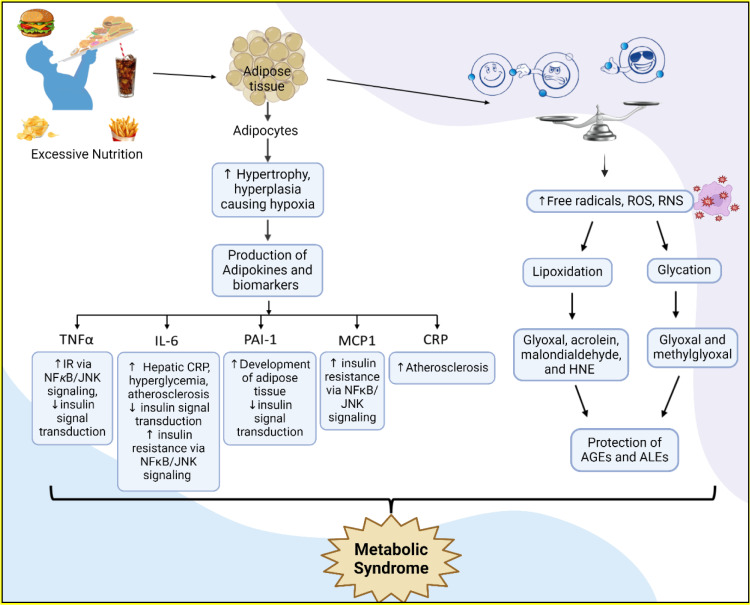
Impact of inflammatory and oxidative mediators on the progression of MetS

Oxidative mediators, which results from the disproportion between oxidants as well as antioxidants, play a significant role in onset and progression of various pathophysiological conditions, including endothelial dysfunction, hypertension, and atherosclerotic cardiovascular diseases (Netzer et al., 2015[124]). In obesity, excess free radicals and oxidants like ROS and reactive nitrogen species (RNS) leading to the oxidation of glucose and lipids, processes known as lipoxidation and glycation, respectively (Dandona et al., 2010[38]). Lipid oxidation results generates lipoxidation compound such as glyoxal, acrolein, malondialdehyde, and 4-hydroxy-nonenal (HNE) (Aldini et al., 2007[2]). These products are highly reactive and contribute to cellular and tissue damage. Similarly, glucose oxidation generates glycation products such as glyoxal and methylglyoxal, these glycated products are reactive and could modify biomolecules, leading to cellular dysfunction. Both lipoxidation and glycation products can interact with amino groups on the amino acids, leading to the formation of AGEs and advanced lipoxidation end products (ALEs) (Aldini et al., 2007[2]). Which are highly reactive molecules, linked to elevated inflammation and oxidative stress (Montezano et al., 2015[114]), This further promotes to chronic inflammation, endothelial dysfunction, IR, and atherosclerosis, which altogether contributes to the pathogenesis of MetS (Francisqueti et al., 2017[57]) (Figure 6(Fig. 6)). 

**Role in MetS: **In MetS, excessive nutrition causes adipocyte hypertrophy and hyperplasia, leading to hypoxia, macrophage infiltration, and the release of pro-inflammatory adipokines. This dysregulated adipokine production contributes to chronic inflammation and insulin resistance, key features of obesity, hypertension, dyslipidemia, and hyperglycemia (Kaplan and Frishman, 2001[80]; Lee and Pratley, 2005[98]; Nishimura et al., 2009[128]). An imbalance of reactive oxygen and nitrogen species aggravates these conditions by promoting lipid and glucose oxidation, forming reactive products like AGEs and ALEs. These molecules further enhance inflammation and oxidative stress, driving the development of endothelial dysfunction, atherosclerosis, with other MetS-associated complications, causing development of MetS (Montezano et al., 2015[114]; Francisqueti et al., 2017[57]). 

### Peroxisome proliferator activator receptors 

PPARs are part of nuclear hormone receptor and transcription factor superfamily. They contains five critical domains (I): the N-terminal A/B domain which includes ligand-independent activation function-1 (AF1) for receptor phosphorylation, (II) a conserved DNA-binding domain (DBD) featuring zinc finger motifs for binding to PPREs, (III) a variable D domain responsible for co-factor interaction, (IV) the E region which contains the ligand binding domain (LBD) responsible for ligand specificity, receptor activation, and dimerization with RXR, and (V) the c-terminal F domain which includes ligand-dependent activation function-2 (AF2) which is essential for co-activator recruitment. The DBD and LBD of human PPARβ/δ and PPARγ show specific amino acid identity percentages compared to human PPARα. 

PPARs has three subtypes, which are classified as PPARα (NR1C1), PPARβ/δ (NR1C2), and PPARγ (NR1C3) (Desvergne and Wahli, 1999[44]; Fajas et al., 2001[52]). The activity of PPAR isoforms is regulated by intricate signaling pathways under different physiological conditions. Disruption in the regulation of these proteins can result in a range of metabolic disorders, including insulin resistance, obesity, hypertension, dyslipidemia, and fatty liver disease. PPARs exert their effects predominantly through a ligand-dependent transactivation mechanism, modulating the transcription of their target genes (Guan and Breyer, 2001[66]). PPARα is primarily found in tissues with high catabolic rates of fatty acids metabolism (like muscles, liver, adipose tissue, renal cortex, and heart), It regulates fatty acid β-oxidation in kidneys, contributing to lower circulating triglyceride levels and elevated HDL cholesterol (Sugden et al., 2001[165]). A lack of PPARα worsen diabetic nephropathy; PPARβ/δ is expressed ubiquitously and is involved in regulating fatty acid oxidation, and energy uncoupling in skeletal muscles, leading to increased energy expenditure, reduced plasma triglycerides, and elevated HDL levels. PPARγ, primarily expresses in adipose tissue, improves insulin sensitivity, reduces TNF-α expression, and enhances adiponectin expression (Sugii and Evans, 2011[166]). It also increases fatty acid uptake, while reducing lipolysis (Ruan et al., 2008[150]), and may exhibit anti-diabetic, anti-inflammatory and anti-atherosclerotic effects (Leone et al., 1999[99]).

**Figure 7:**
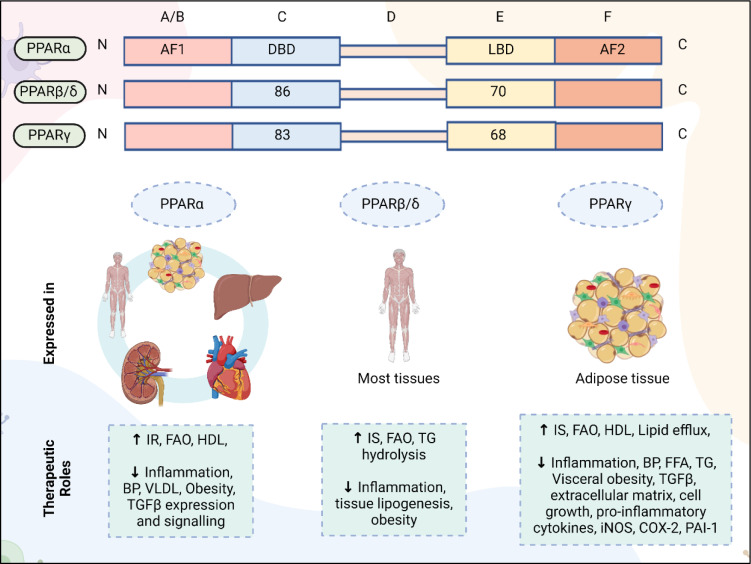
PPAR-mediated therapeutic strategies for MetS

PPARs also modulate the transcription of genes via interacting with various transcription pathways like AP-1, C/EBP, NF-ԟB, and STAT, influencing the cytokines-responsive gene expression. Through the inhibition of these genes, PPARs can decrease cell migration and recruitment of inflammatory cells, which contribute to thrombosis and vasoconstriction (Guan, 2004[65]). In a study on insulin-resistant rhesus monkeys, treatment with PPARβ/δ agonists showed significant results, including increased HDL cholesterol, reduced LDL and triglycerides, with improved fasting insulin levels (Oliver et al., 2001[132]) (Figure 7(Fig. 7)). 

**Role in MetS: **PPARs being part of nuclear hormone receptor family, plays crucial role in regulating MetS. PPARs consist of three subtypes each with distinct tissue distributions and functions. PPARα, predominantly found in tissues involved in fatty acid metabolism, such as the heart and liver, promotes fatty acid β-oxidation, reduces triglyceride levels, and elevates HDL, thereby alleviating dyslipidemia (Sugden et al., 2001[165]). PPARβ/δ, expressed in various tissues, regulates fatty acid oxidation and energy expenditure, contributing to reduced plasma triglycerides and improved lipid profiles, both having roles in preventing obesity (Leone et al., 1999[99]; Ruan et al., 2008[150]). PPARγ, primarily in adipose tissue, improves insulin sensitivity, decreases TNF-α expression, and enhances adiponectin levels, addressing insulin resistance and inflammation (Leone et al., 1999[99]; Ruan et al., 2008[150]). PPARs also modulate gene transcription through interference with pathways like NF-κB and AP-1, reducing inflammatory responses. The therapeutic potential of PPAR agonists has improved lipid profiles and insulin sensitivity in insulin-resistant models, highlighting their relevance in managing MetS (Sugii and Evans, 2011[166]). 

### Gut microbiota 

Gut microbiota is the term for the vast array of microorganisms that inhabit the digestive system. The human gut contains approximately 10^14 ^bacteria, weighing around 1.5 kg, which comprises of 2,000 different species, with a significant majority being anaerobic bacteria (D'Aversa et al., 2013[36]). Among these, approximately 50 bacterial phyla exists, with five major phyla predominating in the gut microbiota. Firmicutes and Actinobacteria are gram-positive, while Verrucomicrobia, Bacteroidetes, and Proteobacteria are gram-negative (Kau et al., 2011[82]). 

Firmicutes represent the largest bacterial phylum in the gut microbiota, playing a key role in breakdown of complex carbohydrates, which enhances energy absorption from food. They also aid in fibres digestion, generating short-chain fatty acids such as butyrate, acetate and propionate, which acts as an energy source for gut cells. The enhanced energy extracted from food can contribute to weight gain. Additionally, Firmicutes aid in maintaining immune tolerance, prevent inflammation and safeguard the entry of pathogens and toxins into the bloodstream (Flint et al., 2012[56]; Tremaroli and Bäckhed, 2012[172]). Bacteroidetes account for approximately 20 genera and has significant role in breakdown of complex polysaccharides, producing metabolic products like lipopolysaccharides, producing metabolic products like lipopolysaccharides (LPS) and *Bacteroides fragilis*. These products can influence the gut-brain axis and central nervous system (CNS) functions (Wexler and Goodman, 2017[182]). Bacteroidetes also regulate immune response through cytokine production and activation of Th17 lymphocytes. In germ-free animals, the development of the immune system is promoted by the capsular polysaccharide component PSA from *B. fragilis *(Ivanov et al., 2008[76]). The genetic capacity of Bacteroides for polysaccharide degradation is facilitated by polysaccharide utilization loci (PUL), which also influence polysaccharide synthesis and responsiveness to dietary changes (Lee et al., 2013[96]). Actinobacteria includes beneficial bacteria like Bifidobacterium, which is a probiotic strain. These bacteria improve gut health by promoting balanced microbiota and enhanced immune functions. Verrucomicrobia includes Akkermansia, which is known to degrade mucous and is essential to maintaining the integrity of the gut lining and promoting gut health (Hou et al., 2022[73]). 

Increased food intake with reduced physical activity can cause alterations in the gut microbiota, leading to an imbalance in various microbial populations (Turnbaugh et al., 2006[173]). For example, the microbiota in obese individuals exhibit an increased capacity for extracting energy, storage it as triglycerides, and utilization through fatty acid oxidation. This microbiota composition regulates various gene expressions and modulates several metabolic pathways, influencing overall metabolic health. The gut microbiota regulates three main metabolic processes (Bäckhed et al., 2004[5]): (1) Polysaccharide degradation, which involves a complex polysaccharide degraded to monosaccharides, facilitated by genes enriched in the obese microbiota. These genes are involved in transporting proteins and fermentation enzymes (Jandhyala et al., 2015[77]). The elevated concentration of fermentation end products serves as an energy source and acts as signalling molecules, regulating energy extraction via receptors like GPR_41_ and GPR_43_, primarily expressed in intestinal enteroendocrine cells; (2) Bile acid metabolism: The composition of bile acid is essential for the solubilization and absorption of dietary fats and fat-soluble vitamins, which are influenced by gut microbiota. Bile acid also acts as a signalling molecule, engaging with receptors like FXR, which stores hepatic fat and lipoperoxidation, and TGR5, which increases GLP-1 release and improves liver and pancreatic functions (Thursby and Juge, 2017[170]); (3) Trimethylamine production: Gut microbiota derived trimethylamine, which is produced from dietary choline found in foods like eggs and red meat, is a proatherogenic compound that links microflora alterations in the intestines to cardiovascular risk. Choline is crucial for cell membranes and lipid metabolism (Zeisel and Da Costa, 2009[188]; Tremaroli and Bäckhed, 2012[172]; Chalotra et al., 2024[20]). 

**Figure 8:**
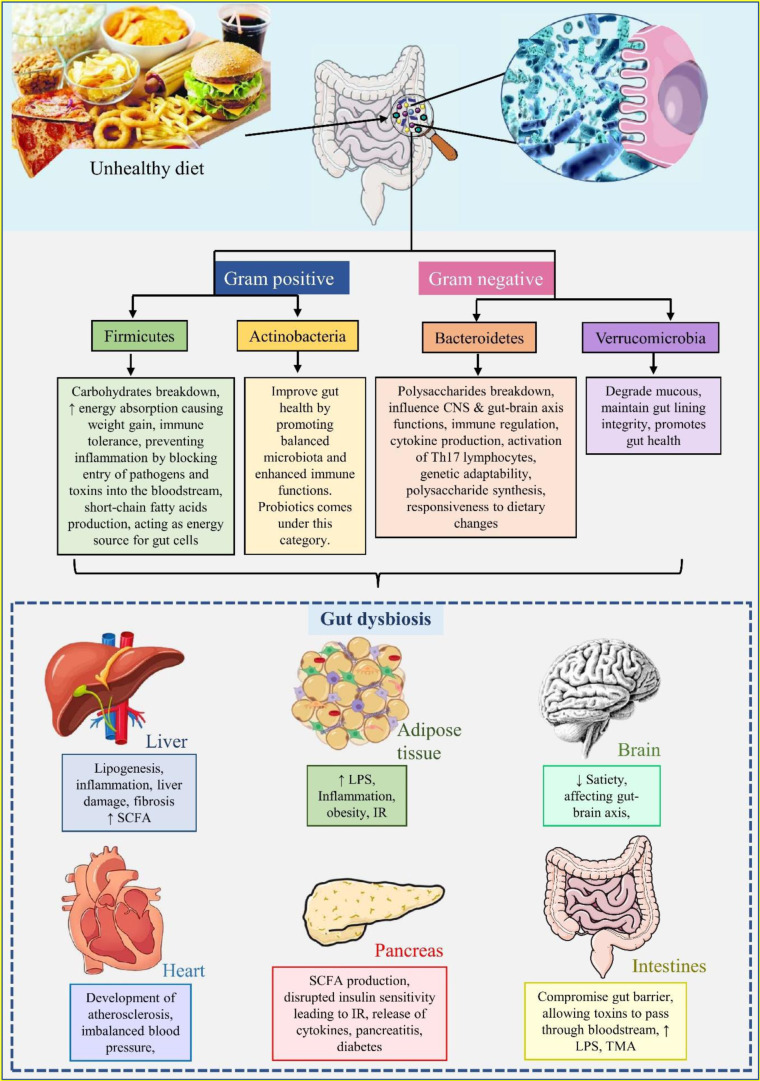
Therapeutic roles of gut microbiota

The gut microbiota also regulates gut permeability, associated with low-grade inflammation characterised by obesity and metabolic disorders (Cani et al., 2009[16]). The endocannabinoid system and GLP-2 signalling are two independent mechanisms involved in gut permeability. Excessive use of antibiotics is a common cause of microbiota imbalance. A study by Bastings et al., has reported gut microbiota's role in satiety regulation (Bastings et al., 2023[9]). For management prebiotics, probiotics, and non-absorbable antibiotics like rifaximin are recommended to restore gut microflora (Ojetti et al., 2009[129]; Delzenne et al., 2011[42], Chalotra et al., 2024[21]) (Figure 8(Fig. 8)). 

**Role in MetS: **The gut microbiota significantly influences metabolic health, plays a crucial role in metabolic health, key bacterial phyla like *Firmicutes* and *Bacteroidetes* regulate energy extraction, fat storage, and immune responses, with an increased *Firmicutes*-to-*Bacteroidetes* ratio linked to obesity and related MetS components (Magne et al., 2020[105]). Gut microbiota influences three primary metabolic processes: polysaccharide degradation, bile acid metabolism, and trimethylamine production, all of which impact energy balance, lipid metabolism, and cardiovascular risk (Thursby and Juge, 2017[170]). Moreover, dysbiosis, or microbial imbalance, disrupts gut permeability, contributing to low-grade inflammation, insulin resistance, and hypertension (Cani et al., 2009[16]). The modulation of gut microbiota through diet, probiotics, and targeted antibiotics holds potential for managing MetS and its associated complications.

Furthermore, additional targets have been identified for further exploration, demonstrating potential in managing various metabolic conditions. Some of these targets include **DPP4:** Dipeptidyl Peptidase 4, an adipokine when an increase in fat cells and smooth and skeletal muscle cells impairs insulin signalling. In visceral fat patients, there was a five-times increase in DPP4 compared to subcutaneous fat. With the reduction in body weight, there is two times decrease in DPP4 levels. DPP4 release correlates with adipocyte size and is a potential source of its release, linking it with metabolic syndrome (Lamers et al., 2011[94]); **MCH-1R:** Melanin concentrating hormone-1 Receptor, a G-protein coupled receptor, also known as somatostatin receptor like protein or G-protein couple receptor 24. MCH-1R protein is found in rodents, and higher mammalian species, expressed in various parts of brain, also associated with olfaction, feeding behaviour, and energy homeostasis (Kowalski and McBriar, 2004[91]); **GPR119: **It is a receptor, that belongs to GPCR family that operates through two mechanisms: it enhances insulin secretion from the pancreas and triggers the release of incretins from the intestines, These incretins are key in modulating insulin secretion, promoting feeling of satiety, and regulating glucagon secretion (Yang et al., 2018[186]); **Glucokinase regulatory protein: **These are small molecules which inhibits the interaction between glucokinase and its regulatory proteins. Genome wide association studies have identified glucokinase and its regulatory protein as potential targets for managing T II DM, NAFLD, and dyslipidemia; **AT1 receptors**: Angiotensin I receptor antagonist has shown anti-obesity effects and restores leptin sensitivity (Müller‐Fielitz et al., 2014[119]); **FXR**: The Farnesoid X Receptor is a nuclear receptor for bile acid that acts as a transcription factor, modulating the expression of specific targeted genes. It is highly expressed in the intestines, liver, adipose tissue, kidney and adrenal glands. It helps protect liver cells from bile acids. It improves insulin signalling and insulin-induced glucose uptake in adipocytes (Cariou et al., 2006[18]). It has reported roles in glucose homeostasis, lipid metabolism, atherosclerosis (Cariou and Staels, 2007[17]); **sEH:** Soluble epoxide hydrolase modulates the inflammatory condition. Elevated sEH activity in adipose tissue leads to low-grade inflammation promoting MetS (Bah et al., 2024[6]). These targets and the molecular targets mentioned in this manuscript could be either targeted alone or in combination to mitigate all possible conditions associated with the therapeutic management of MetS. 

## Future Perspective and Conclusion

Management of MetS remains a complex challenge influenced by various physiological, genetic, molecular, and environmental factors. Conditions like hyperglycemia, dyslipidemia, hypertension, visceral obesity, oxidative stress, inflammation, and gut dysbiosis play central roles in the development of MetS (Yamaoka and Tango, 2012[185]). Excessive phosphate intake also contributes to MetS by promoting inflammation, vascular stiffness, and hypertension, further contributing to metabolic disturbances like insulin resistance, diabetes, and obesity. Gut dysbiosis also influences absorption, fat storage, and immune responses, playing a significant role in MetS progression and linking microbial imbalance to obesity, insulin resistance, and cardiovascular risks. 

The interaction of these diseases amplifies the severity of the syndrome, necessitating a multifaceted approach which targets multiple underlying conditions. Recent advancements have unveiled new therapeutic targets that hold the potential to manage MetS including PPAR, leptin, asprosin, gut microbiota, and signalling pathways like AMPK. AMPK signalling pathway has a complex network of pharmacological roles; it also involves the regulation of various metabolic conditions and fatty acid pathways, including ROS (Srivastava et al., 2012[163]; Bullon et al., 2016[15]). GLP-1 receptor agonists have demonstrated a role in type-II diabetes and obesity management by enhancing insulin secretion, promoting weight loss, and regulating cardiovascular, renal, and hepatic functions (Sattar et al., 2021[153]). SGLT2 inhibitors help lower blood glucose levels, mild to moderate body fat or obesity, with blood pressure. The effects of obesity are not so prominent, so combination with drugs like GLP-1 agonist, shows greater benefits in conditions like obesity, atherosclerotic, cardiovascular disease and heart failure (Pereira and Eriksson, 2019[135]). Leptin a key regulator of appetite, metabolism, and energy expenditure, gets dysregulated in obesity and type II diabetes, leading to leptin resistance, which leads to the development and progression of MetS. Targeting leptin resistance can restore energy balance, and improves insulin sensitivity. Similarly, asprosin, an adipokine that plays role in appetite, inducing insulin resistance and inflammation, contributing to obesity and metabolic dysregulation in MetS; but research on its therapeutic modulation remains limited or unexplored yet. PPARs with their three subtypes, are crucial for regulating fatty acid metabolism, which helps to regulate fatty acid metabolism, insulin sensitivity, and inflammation, making them key targets for managing metabolic syndrome (MetS) by improving lipid profiles, reducing insulin resistance, and controlling inflammation. Additionally, targeting specific molecular pathways involved in inflammation and oxidative stress mechanisms could further alleviate the conditions associated with MetS.

Future research should prioritise validating these targets in clinical studies and developing specific inhibitors or activators to modulate their activity effectively. Advancements in genomics and metabolomics enable the identification of biomarkers that predict the molecular mechanisms through individual responses to therapy and guide personalised treatment strategies. Using molecular-targeted medications alone or combined with other therapeutic approaches could markedly improve the chances of developing an effective treatment strategy for MetS. Moreover, the development of selective drugs and the exploration of emerging molecular targets like MCH-1R, glucokinase regulatory proteins, sEH, GPR119, and FXR offer new therapeutic opportunities to address obesity, insulin resistance, inflammation, and dyslipidemia, which are hallmark features of MetS. Ultimately, integrating ongoing research on molecular targets with clinical studies will establish a comprehensive scientific foundation necessary for developing a wide range of therapeutic strategies, allowing for a safer, effective approach that targets the multifaceted nature of this complex disorder. 

## Declaration

### Conflict of interest

The authors declare no conflict of interest.

### Artificial Intelligence (AI) - Assisted Technology

The authors acknowledge the use to AI (ChatGPT) to assist in improving the clarity and readability of the manuscript, which were later reviewed and verified by the authors. 

### Funding 

No funding was received.

## Figures and Tables

**Figure 1:**
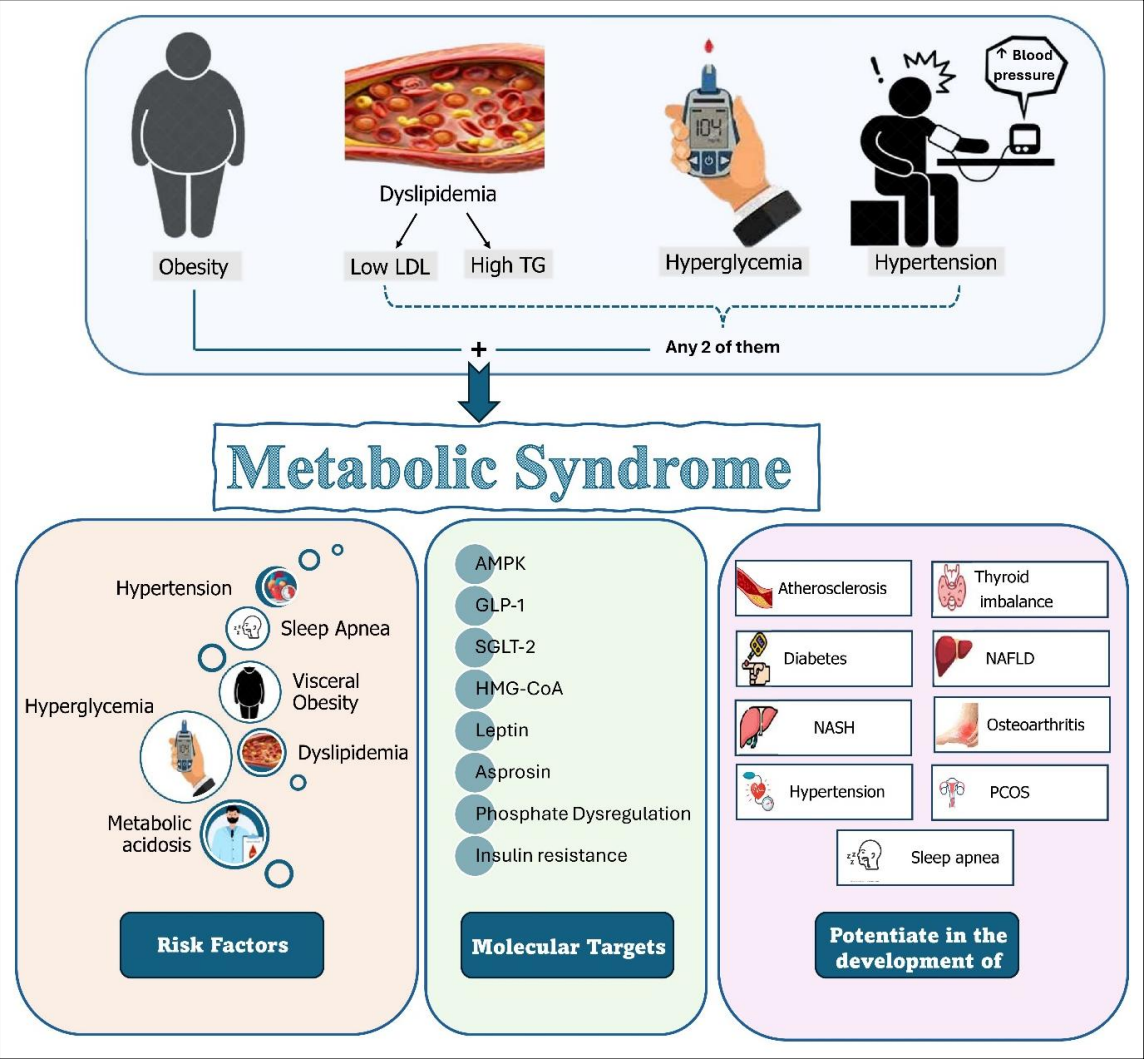
Graphical abstract

## References

[1] Alberti KGM, Zimmet P, Shaw J (2005). The metabolic syndrome—a new worldwide definition. The Lancet.

[2] Aldini G, Dalle‐Donne I, Facino RM, Milzani A, Carini M (2007). Intervention strategies to inhibit protein carbonylation by lipoxidation‐derived reactive carbonyls. Medicinal Research Reviews.

[3] Anderson TJ, Meredith IT, Yeung AC, Frei B, Selwyn AP, Ganz P (1995). The effect of cholesterol-lowering and antioxidant therapy on endothelium-dependent coronary vasomotion. New England Journal of Medicine.

[4] Athyros VG, Mikhailidis DP (2015). High incidence of metabolic syndrome further increases cardiovascular risk in patients with type 2 diabetes. Implications for everyday practice. Journal of Diabetes and its Complications.

[5] Bäckhed F, Ding H, Wang T, Hooper LV, Koh GY, Nagy A (2004). The gut microbiota as an environmental factor that regulates fat storage. Proceedings of the National Academy of Sciences of the U S A.

[6] Bah TM, Davis CM, Allen E, Borkar RN, Perez R, Grafe MR (2024). Soluble Epoxide Hydrolase Inhibition Reverses Cognitive Dysfunction in a Mouse Model of Metabolic Syndrome by Modulating Inflammation. Prostaglandins & Other Lipid Mediators.

[7] Bansal AB, Cassagnol M (2019). HMG-CoA Reductase Inhibitors.

[8] Bansal N (2015). Prediabetes diagnosis and treatment: A review. World Journal of Diabetes.

[9] Bastings JJ, Venema K, Blaak EE, Adam TC (2023). Influence of the gut microbiota on satiety signaling. Trends in Endocrinology & Metabolism.

[10] Bays H, Abate N, Chandalia M (2005). Adiposopathy: sick fat causes high blood sugar, high blood pressure and dyslipidemia. Future Cardiology.

[11] Beaupere C, Liboz A, Fève B, Blondeau B, Guillemain G (2021). Molecular mechanisms of glucocorticoid-induced insulin resistance. International Journal of Molecular Sciences.

[12] Belhayara MI, Mellouk Z, Hamdaoui MS, Bachaoui M, Kheroua O, Malaisse WJ (2019). Relationship between the insulin resistance and circulating predictive biochemical markers in metabolic syndrome among young adults in western Algeria. Diabetes & Metabolic Syndrome: Clinical Research & Reviews.

[13] Bhagavan N, Ha C-E, Ha C-E, Bhagavan NV (2011). Lipids II: Phospholipids, glycosphingolipids, and cholesterol. Essentials of Medical Biochemistry. With Clinical Cases (chapter 17).

[14] Bhalwar R (2020). Metabolic syndrome: The Indian public health perspective. Medical Journal Armed Forces India.

[15] Bullon P, Marin-Aguilar F, Roman-Malo L (2016). AMPK/Mitochondria in Metabolic Diseases. Exp Suppl.

[16] Cani PD, Possemiers S, Van de Wiele T, Guiot Y, Everard A, Rottier O (2009). Changes in gut microbiota control inflammation in obese mice through a mechanism involving GLP-2-driven improvement of gut permeability. Gut.

[17] Cariou B, Staels B (2007). FXR: a promising target for the metabolic syndrome?. Trends in Pharmacological Sciences.

[18] Cariou B, Van Harmelen K, Duran-Sandoval D, Van Dijk TH, Grefhorst A, Abdelkarim M (2006). The farnesoid X receptor modulates adi-posity and peripheral insulin sensitivity in mice. Journal of Biological Chemistry.

[19] Celotto A, Ferreira L, Capellini V, Albuquerque A, Rodrigues AJ, Evora PRB (2016). Acute but not chronic metabolic acidosis potentiates the acetylcholine-induced reduction in blood pressure: an endothelium-dependent effect. Brazilian Journal of Medical and Biological Research.

[20] Chalotra R, Amanat M, Dhanawat M, Singh R, Gupta N, Dhanawat M (2024). Oleanolic Acid and Its Derivatives: Therapeutic Potential in Bacterial Infections. Properties and Uses of Oleanolic Acid.

[21] Chalotra R, Chaudhary K, Dhankhar S, Chauhan S, Dhanawat M, Gupta S (2024). Nutraceutical - An Alternative Pathway in Therapeutics. Anthocyanins: Pharmacology and Nutraceutical Importance.

[22] Chalotra R, Dhanawat M, Chauhan S, Mujwar S, Gupta S (2024). Evaluation of Iris Kashmiriana Baker plant extracts against nociception and rheumatoid arthritis in experimental rats: A concept proof by In-silico model. Journal of Ethnopharmacology.

[23] Chalotra R, Gupta T, Chib S, Amanat M, Kumar P, Singh R (2024). Treatment of diabetic complications: do flavonoids holds the keys?. Critical Reviews in Food Science and Nutrition.

[24] Chang AR, Lazo M, Appel LJ, Gutierrez OM, Grams ME (2014). High dietary phosphorus intake is associated with all-cause mortality: results from NHANES III. The American Journal of Clinical Nutrition.

[25] Chao EC, Henry RR (2010). SGLT2 inhibition—a novel strategy for diabetes treatment. Nature Reviews Drug Discovery.

[26] Chassaing B, Gewirtz AT (2014). Gut microbiota, low-grade inflammation, and metabolic syndrome. Toxicologic Pathology.

[27] Chauhan S, Chalotra R, Rathi A, Saini M, Deol S, Lard M (2023). Current approaches in healing of wounds in diabetes and diabetic foot ulcers. Current Bioactive Compounds.

[28] Chimonas T, Karagiannis A, Athyros VG, Achimastos A, Elisaf M, Panagiotakos DB (2010). Blood pressure levels constitute the most important determinant of the metabolic syndrome in a Mediterranean population: a discrimination analysis. Metabolic Syndrome and Related Disorders.

[29] Choi SM, Tucker DF, Gross DN, Easton RM, DiPilato LM, Dean AS (2010). Insulin regulates adipocyte lipolysis via an Akt-independent signaling pathway. Molecular and Cellular Biology.

[30] Chokroverty S (2010). Overview of sleep & sleep disorders. Indian Journal of Medical Research.

[31] Ciccone M, Vettor R, Pannacciulli N, Minenna A, Bellacicco M, Rizzon P (2001). Plasma leptin is independently associated with the intima-media thickness of the common carotid artery. International Journal of Obesity.

[32] Coelho M, Oliveira T, Fernandes R (2013). State of the art paper Biochemistry of adipose tissue: an endocrine organ. Archives of Medical Science.

[33] Coll AP, Yeo GS (2013). The hypothalamus and metabolism: integrating signals to control energy and glucose homeostasis. Current Opinion in Pharmacology.

[34] Collins L, Costello RA (2019). Glucagon-like peptide-1 receptor agonists.

[35] Cryer PE (2011). Death during intensive glycemic therapy of diabetes: mechanisms and implications. The American Journal of Medicine.

[36] D’Aversa F, Tortora A, Ianiro G, Ponziani FR, Annicchiarico BE, Gasbarrini A (2013). Gut microbiota and metabolic syndrome. Internal and Emergency Medicine.

[37] Dandona P, Aljada A, Bandyopadhyay A (2004). Inflammation: the link between insulin resistance, obesity and diabetes. Trends in Immunology.

[38] Dandona P, Ghanim H, Chaudhuri A, Dhindsa S, Kim SS (2010). Macronutrient intake induces oxidative and inflammatory stress: potential relevance to atherosclerosis and insulin resistance. Experimental & Molecular Medicine.

[39] Davidson MH (2011). Cardiovascular effects of glucagonlike peptide–1 agonists. The American Journal of Cardiology.

[40] de Candia P, Prattichizzo F, Garavelli S, Alviggi C, La Cava A, Matarese G (2021). The pleiotropic roles of leptin in metabolism, immunity, and cancer. Journal of Experimental Medicine.

[41] DeFronzo RA (2009). From the triumvirate to the ominous octet: a new paradigm for the treatment of type 2 diabetes mellitus. Diabetes.

[42] Delzenne NM, Neyrinck AM, Bäckhed F, Cani PD (2011). Targeting gut microbiota in obesity: effects of prebiotics and probiotics. Nature Reviews Endocrinology.

[43] Després J-P, Lemieux I (2006). Abdominal obesity and metabolic syndrome. Nature.

[44] Desvergne B, Wahli W (1999). Peroxisome proliferator-activated receptors: nuclear control of metabolism. Endocrine Reviews.

[45] Di Chiara T, Argano C, Corrao S, Scaglione R, Licata G (2012). Hypoadiponectinemia: a link between visceral obesity and metabolic syndrome. Journal of Nutrition and Metabolism.

[46] Du X, Edelstein D, Obici S, Higham N, Zou M-H, Brownlee M (2006). Insulin resistance reduces arterial prostacyclin synthase and eNOS activities by increasing endothelial fatty acid oxidation. The Journal of Clinical Investigation.

[47] Duggal DD (2022). Diabetes Management [Internet]. Dr. Surajeet Kumar Patra M, MD, FDIAB, MBA & APMP, editor. Online Breathe Well-Being. https://www.breathewellbeing.in/blog/hyperinsulinemia-signs-causes-diagnosis-treatment-diet/.

[48] Duncan BB, Schmidt MI, Pankow JS, Ballantyne CM, Couper D, Vigo A (2003). Low-grade systemic inflammation and the development of type 2 diabetes: the atherosclerosis risk in communities study. Diabetes.

[49] Eckel R, Yost T, Jensen D (1995). Alterations in lipoprotein lipase in insulin resistance. International Journal of Obesity and Related Metabolic Disorders.

[50] Eckel RH, Grundy SM, Zimmet PZ (2005). The metabolic syndrome. The Lancet.

[51] Fahed G, Aoun L, Bou Zerdan M, Allam S, Bou Zerdan M, Bouferraa Y (2022). Metabolic syndrome: updates on pathophysiology and management in 2021. International Journal of Molecular Sciences.

[52] Fajas L, Debril M, Auwerx J (2001). Peroxisome proliferator-activated receptor-gamma: from adipogenesis to carcinogenesis. Journal of Molecular Endocrinology.

[53] Farrag M, Ait Eldjoudi D, González-Rodríguez M, Cordero-Barreal A, Ruiz-Fernández C, Capuozzo M (2023). Asprosin in health and disease, a new glucose sensor with central and peripheral metabolic effects. Frontiers in Endocrinology.

[54] Fletcher EC (1995). The relationship between systemic hypertension and obstructive sleep apnea: facts and theory. The American Journal of Medicine.

[55] Flier JS, Harris M, Hollenberg AN (2000). Leptin, nutrition, and the thyroid: the why, the wherefore, and the wiring. The Journal of Clinical Investigation.

[56] Flint HJ, Scott KP, Duncan SH, Louis P, Forano E (2012). Microbial degradation of complex carbohydrates in the gut. Gut Microbes.

[57] Francisqueti FV, Chiaverini LCT, Santos KCd, Minatel IO, Ronchi CB, Ferron AJT (2017). The role of oxidative stress on the pathophysiology of metabolic syndrome. Revista da Associação Médica Brasileira.

[58] Furnary AP, Wu Y (2006). Clinical effects of hyperglycemia in the cardiac surgery population: the Portland Diabetic Project. Endocrine Practice.

[59] Gallwitz B (2011). GLP-1 agonists and dipeptidyl-peptidase IV inhibitors. Handb Exp Pharmacol.

[60] Gami AS, Caples SM, Somers VK (2003). Obesity and obstructive sleep apnea. Endocrinology and Metabolism Clinics.

[61] Garber AJ (2011). Long-acting glucagon-like peptide 1 receptor agonists: a review of their efficacy and tolerability. Diabetes Care.

[62] Gharaibeh NE, Rahhal MN, Rahimi L, Ismail-Beigi F (2019). SGLT-2 inhibitors as promising therapeutics for non-alcoholic fatty liver disease: pathophysiology, clinical outcomes, and future directions. Diabetes Metab Syndr Obes.

[63] González-Regueiro JA, Moreno-Castañeda L, Uribe M, Chávez-Tapia NC (2018). The role of bile acids in glucose metabolism and their relation with diabetes. Annals of Hepatology.

[64] Greig FH, Ewart M-A, McNaughton E, Cooney J, Spickett CM, Kennedy S (2015). The hypotensive effect of acute and chronic AMP-activated protein kinase activation in normal and hyper-lipidemic mice. Vascular Pharmacology.

[65] Guan Y (2004). Peroxisome proliferator-activated receptor family and its relationship to renal complications of the metabolic syndrome. Journal of the American Society of Nephrology.

[66] Guan Y, Breyer MD (2001). Peroxisome proliferator-activated receptors (PPARs): novel therapeutic targets in renal disease. Kidney International.

[67] Gupta A, Chalotra R, Singh R (2025). Preclinical Evaluation of Fisetin in the Management of Diabetic Foot Ulcer in Wistar Rats. Curr Diabetes Rev.

[68] Gutzwiller J-P, Hruz P, Huber AR, Hamel C, Zehnder C, Drewe J (2006). Glucagon-like peptide-1 is involved in sodium and water homeostasis in humans. Digestion.

[69] Halaas JL, Gajiwala KS, Maffei M, Cohen SL, Chait BT, Rabinowitz D (1995). Weight-reducing effects of the plasma protein encoded by the obese gene. Science.

[70] Hinnen D (2017). Glucagon-like peptide 1 receptor agonists for type 2 diabetes. Diabetes Spectrum.

[71] Holmes BF, Kurth-Kraczek E, Winder W (1999). Chronic activation of 5′-AMP-activated protein kinase increases GLUT-4, hexokinase, and glycogen in muscle. Journal of Applied Physiology.

[72] Horike N, Sakoda H, Kushiyama A, Ono H, Fujishiro M, Kamata H (2008). AMP-activated protein kinase activation increases phosphorylation of glycogen synthase kinase 3β and thereby reduces cAMP-responsive element transcriptional activity and phosphoenolpyruvate carboxykinase C gene expression in the liver. Journal of Biological Chemistry.

[73] Hou K, Wu Z-X, Chen X-Y, Wang J-Q, Zhang D, Xiao C (2022). Microbiota in health and diseases. Signal Transduction and Targeted Therapy.

[74] Hurrle S, Hsu WH (2017). The etiology of oxidative stress in insulin resistance. Biomedical Journal.

[75] Ichikawa S, Sorenson AH, Austin AM, Mackenzie DS, Fritz TA, Moh A (2009). Ablation of the Galnt3 gene leads to low-circulating intact fibroblast growth factor 23 (Fgf23) concentrations and hyperphosphatemia despite increased Fgf23 expression. Endocrinology.

[76] Ivanov II, de Llanos Frutos R, Manel N, Yoshinaga K, Rifkin DB, Sartor RB (2008). Specific microbiota direct the differentiation of IL-17-producing T-helper cells in the mucosa of the small intestine. Cell Host Microbe.

[77] Jandhyala SM, Talukdar R, Subramanyam C, Vuyyuru H, Sasikala M, Reddy DN (2015). Role of the normal gut microbiota. World Journal of Gastroenterology.

[78] Janečková R (2001). The role of leptin in human physiology and pathophysiology. Physiological Research.

[79] Jensen MD, Caruso M, Heiling V, Miles JM (1989). Insulin regulation of lipolysis in nondiabetic and IDDM subjects. Diabetes.

[80] Kaplan RC, Frishman WH (2001). Systemic inflammation as a cardiovascular disease risk factor and as a potential target for drug therapy. Heart Disease (Hagerstown, Md).

[81] Katsimardou A, Imprialos K, Stavropoulos K, Sachinidis A, Doumas M, Athyros V (2020). Hypertension in metabolic syndrome: novel insights. Current Hypertension Reviews.

[82] Kau AL, Ahern PP, Griffin NW, Goodman AL, Gordon JI (2011). Human nutrition, the gut microbiome and the immune system. Nature.

[83] Kaye WH, Gendall K, Kye C (1998). The role of the central nervous system in the psychoneuroendocrine disturbances of anorexia and bulimia nervosa. Psychiatric Clinics.

[84] Kaye WH, Klump K, Frank G, Strober M (2000). Anorexia and bulimia nervosa. Annual Review of Medicine.

[85] Khan K, Alkatsha A, Sarhan M, Abuhamar F, Abdulrzaq R, Khan A (2020). Evaluation of drug interactions with medications prescribed to ambulatory patients with metabolic syndrome in urban area. Adv Mater Lett.

[86] Kolb H, Kempf K, Röhling M, Lenzen-Schulte M, Schloot NC, Martin S (2021). Ketone bodies: from enemy to friend and guardian angel. BMC Medicine.

[87] Koo BK, Park S, Han K-D, Moon MK (2021). Hypertriglyceridemia is an independent risk factor for cardiovascular diseases in Korean adults aged 30–49 years: a nationwide population-based study. Journal of Lipid and Atherosclerosis.

[88] Körner M, Stöckli M, Waser B, Reubi JC (2007). GLP-1 receptor expression in human tumors and human normal tissues: potential for in vivo targeting. Journal of Nuclear Medicine.

[89] Kotsis V, Stabouli S, Papakatsika S, Rizos Z, Parati G (2010). Mechanisms of obesity-induced hypertension. Hypertension Research.

[90] Kotsis V, Tsioufis K, Antza C, Seravalle G, Coca A, Sierra C (2018). Obesity and cardiovascular risk: a call for action from the European Society of Hypertension Working Group of Obesity, Diabetes and the High-risk Patient and European Association for the Study of Obesity: part B: obesity-induced cardiovascular disease, early prevention strategies and future research directions. Journal of Hypertension.

[91] Kowalski TJ, McBriar MD (2004). Therapeutic potential of melanin-concentrating hormone-1 receptor antagonists for the treatment of obesity. Expert Opinion on Investigational Drugs.

[92] Kraut JA, Madias NE (2010). Metabolic acidosis: pathophysiology, diagnosis and management. Nature Reviews Nephrology.

[93] Lacerda-Abreu MA, Meyer-Fernandes JR (2021). Extracellular inorganic phosphate-induced release of reactive oxygen species: roles in physiological processes and disease development. International Journal of Molecular Sciences.

[94] Lamers D, Famulla S, Wronkowitz N, Hartwig S, Lehr S, Ouwens DM (2011). Dipeptidyl peptidase 4 is a novel adipokine potentially linking obesity to the metabolic syndrome. Diabetes.

[95] Lee G-H, Proenca R, Montez J, Carroll K, Darvishzadeh J, Lee J (1996). Abnormal splicing of the leptin receptor in diabetic mice. Nature.

[96] Lee SM, Donaldson GP, Mikulski Z, Boyajian S, Ley K, Mazmanian SK (2013). Bacterial colonization factors control specificity and stability of the gut microbiota. Nature.

[97] Lee Y, Siddiqui WJ (2019). Cholesterol levels.

[98] Lee Y-H, Pratley RE (2005). The evolving role of inflammation in obesity and the metabolic syndrome. Current Diabetes Reports.

[99] Leone TC, Weinheimer CJ, Kelly DP (1999). A critical role for the peroxisome proliferator-activated receptor α (PPARα) in the cellular fasting response: the PPARα-null mouse as a model of fatty acid oxidation disorders. Proceedings of the National Academy of Sciences of the U S A.

[100] Lin Y-C, Huang J, Hileman S, Martin KH, Hull R, Davis M (2015). Leptin decreases heart rate associated with increased ventricular repolarization via its receptor. American Journal of Physiology-Heart and Circulatory Physiology.

[101] Liu Y, Wei X, Wu M, Xu J, Xu B, Kang L (2021). Cardioprotective roles of β-hydroxybutyrate against doxorubicin induced cardiotoxicity. Frontiers in Pharmacology.

[102] Lo J, Bernstein LE, Canavan B, Torriani M, Jackson MB, Ahima RS (2007). Effects of TNF-α neutralization on adipocytokines and skeletal muscle adiposity in the metabolic syndrome. American Journal of Physiology-Endocrinology and Metabolism.

[103] Loh K, Tam S, Murray‐Segal L, Huynh K, Meikle PJ, Scott JW (2019). Inhibition of Adenosine Monophosphate–Activated Protein Kinase–3‐Hydroxy‐3‐Methylglutaryl Coenzyme A Reductase Signaling Leads to Hypercholesterolemia and Promotes Hepatic Steatosis and Insulin Resistance. Hepatology Communications.

[104] Maedler K, Sergeev P, Ris F, Oberholzer J, Joller-Jemelka HI, Spinas GA (2002). Glucose-induced β cell production of IL-1β contributes to glucotoxicity in human pancreatic islets. The Journal of Clinical Investigation.

[105] Magne F, Gotteland M, Gauthier L, Zazueta A, Pesoa S, Navarrete P (2020). The firmicutes/bacteroidetes ratio: a relevant marker of gut dysbiosis in obese patients?. Nutrients.

[106] Mancia G, Bousquet P, Elghozi JL, Esler M, Grassi G, Julius S (2007). The sympathetic nervous system and the metabolic syndrome. Journal of Hypertension.

[107] Mannucci E, Monami M (2017). Cardiovascular safety of incretin-based therapies in type 2 diabetes: systematic review of integrated analyses and randomized controlled trials. Advances in Therapy.

[108] Matsuzawa Y, Funahashi T, Kihara S, Shimomura I (2004). Adiponectin and metabolic syndrome. Arteriosclerosis Thrombosis Vascular Biology.

[109] Mausner-Fainberg K, Luboshits G, Mor A, Maysel-Auslender S, Rubinstein A, Keren G (2008). The effect of HMG-CoA reductase inhibitors on naturally occurring CD4+ CD25+ T cells. Atherosclerosis.

[110] Mendrick DL, Diehl AM, Topor LS, Dietert RR, Will Y, La Merrill MA (2018). Metabolic syndrome and associated diseases: from the bench to the clinic. Toxicological Sciences.

[111] Menuet R, Lavie CJ, Milani RV (2005). Importance and management of dyslipidemia in the metabolic syndrome. The American Journal of the Medical Sciences.

[112] Mironov N, Haque M, Atfi A, Razzaque MS (2022). Phosphate Dysregulation and Metabolic Syndrome. Nutrients.

[113] Misra A, Khurana L (2008). Obesity and the metabolic syndrome in developing countries. The Journal of Clinical Endocrinology & Metabolism.

[114] Montezano AC, Dulak-Lis M, Tsiropoulou S, Harvey A, Briones AM, Touyz RM (2015). Oxidative stress and human hypertension: vascular mechanisms, biomarkers, and novel therapies. Canadian Journal of Cardiology.

[115] Moore JX, Chaudhary N, Akinyemiju T (2017). Metabolic Syndrome Prevalence by Race/Ethnicity and Sex in the United States, National Health and Nutrition Examination Survey, 1988-2012. Prev Chronic Dis.

[116] Morcos YA, Lütke S, Tenbieg A, Hanisch F-G, Pryymachuk G, Piekarek N (2022). Sensitive asprosin detection in clinical samples reveals serum/saliva correlation and indicates cartilage as source for serum asprosin. Scientific Reports.

[117] Mousa A, Naderpoor N, Teede H, Scragg R, de Courten B (2018). Vitamin D supplementation for improvement of chronic low-grade inflammation in patients with type 2 diabetes: a systematic review and meta-analysis of randomized controlled trials. Nutrition Reviews.

[118] Müller S, Martin S, Koenig W, Hanifi-Moghaddam P, Rathmann W, Haastert B (2002). Impaired glucose tolerance is associated with increased serum concentrations of interleukin 6 and co-regulated acute-phase proteins but not TNF-α or its receptors. Diabetologia.

[119] Müller‐Fielitz H, Hübel N, Mildner M, Vogt FM, Barkhausen J, Raasch W (2014). Chronic blockade of angiotensin AT1 receptors improves cardinal symptoms of metabolic syndrome in diet‐induced obesity in rats. British Journal of Pharmacology.

[120] Nakamura T, Tokunaga K, Shimomura I, Nishida M, Yoshida S, Kotani K (1994). Contribution of visceral fat accumulation to the development of coronary artery disease in non-obese men. Atherosclerosis.

[121] Narkar VA, Downes M, Ruth TY, Embler E, Wang Y-X, Banayo E (2008). AMPK and PPARδ agonists are exercise mimetics. Cell.

[122] Nawaz SS, Siddiqui K (2022). Plasminogen activator inhibitor-1 mediate downregulation of adiponectin in type 2 diabetes patients with metabolic syndrome. Cytokine.

[123] Neeland IJ, Ross R, Després J-P, Matsuzawa Y, Yamashita S, Shai I (2019). Visceral and ectopic fat, atherosclerosis, and cardiometabolic disease: a position statement. The Lancet Diabetes & Endocrinology.

[124] Netzer N, Gatterer H, Faulhaber M, Burtscher M, Pramsohler S, Pesta D (2015). Hypoxia, oxidative stress and fat. Biomolecules.

[125] Nguyen TT, Quan X, Hwang K-H, Xu S, Das R, Choi S-K (2015). Mitochondrial oxidative stress mediates high-phosphate-induced secretory defects and apoptosis in insulin-secreting cells. American Journal of Physiology - Endocrinology and Metabolism.

[126] Ni L, Yuan C, Chen G, Zhang C, Wu X (2020). SGLT2i: beyond the glucose-lowering effect. Cardiovascular Diabetology.

[127] Nieto FJ, Young TB, Lind BK, Shahar E, Samet JM, Redline S (2000). Association of sleep-disordered breathing, sleep apnea, and hypertension in a large community-based study. Jama.

[128] Nishimura S, Manabe I, Nagasaki M, Eto K, Yamashita H, Ohsugi M (2009). CD8+ effector T cells contribute to macrophage recruitment and adipose tissue inflammation in obesity. Nature Medicine.

[129] Ojetti V, Lauritano EC, Barbaro F, Migneco A, Ainora ME, Fontana L (2009). Rifaximin pharmacology and clinical implications. Expert Opinion on Drug Metabolism & Toxicology.

[130] Ojo O, Ojo OO, Zand N, Wang X (2021). The effect of dietary fibre on gut microbiota, lipid profile, and inflammatory markers in patients with type 2 diabetes: a systematic review and meta-analysis of randomised controlled trials. Nutrients.

[131] Okerson T, Chilton RJ (2012). The cardiovascular effects of GLP‐1 receptor agonists. Cardiovascular Therapeutics.

[132] Oliver WR, Shenk JL, Snaith MR, Russell CS, Plunket KD, Bodkin NL (2001). A selective peroxisome proliferator-activated receptor δ agonist promotes reverse cholesterol transport. Proceedings of the National Academy of Sciences of the U S A.

[133] Parish JM, Adam T, Facchiano L (2007). Relationship of metabolic syndrome and obstructive sleep apnea. Journal of Clinical Sleep Medicine.

[134] Paz-Filho G, Mastronardi C, Wong M-L, Licinio J (2012). Leptin therapy, insulin sensitivity, and glucose homeostasis. Indian Journal of Endocrinology and Metabolism.

[135] Pereira MJ, Eriksson JW (2019). Emerging role of SGLT-2 inhibitors for the treatment of obesity. Drugs.

[136] Pettersson B, Rosenqvist U, Deleskog A, Journath G, Wändell P (2011). Self-reported experience of hypoglycemia among adults with type 2 diabetes mellitus (Exhype). Diabetes Research and Clinical Practice.

[137] Pihlajamäki J, Gylling H, Miettinen TA, Laakso M (2004). Insulin resistance is associated with increased cholesterol synthesis and decreased cholesterol absorption in normoglycemic men. Journal of Lipid Research.

[138] Pimenta A, Gaidhu M, Habib S, So M, Fediuc S, Mirpourian M (2008). Prolonged exposure to palmitate impairs fatty acid oxidation despite activation of AMP‐activated protein kinase in skeletal muscle cells. Journal of Cellular Physiology.

[139] Pinto J, Garpestad E, Weiss JW, Bergau DM, Kirby DA (1993). Hemodynamic changes associated with obstructive sleep apnea followed by arousal in a porcine model. Journal of Applied Physiology.

[140] Porte D (1999). Mechanisms for Hyperglycemia in the Metabolic Syndrome: The Key Role of β‐Cell Dysfunction. Annals of the New York Academy of Sciences.

[141] Pothiwala P, Jain SK, Yaturu S (2009). Metabolic syndrome and cancer. Metabolic Syndrome and Related Disorders.

[142] Premji R, Nylen ES, Naser N, Gandhi S, Burman KD, Sen S (2022). Lipid profile changes associated with SGLT-2 inhibitors and GLP-1 agonists in diabetes and metabolic syndrome. Metabolic Syndrome and Related Disorders.

[143] Razzaque MS (2011). Phosphate toxicity: new insights into an old problem. Clinical Science.

[144] Razzaque MS (2009). The FGF23–Klotho axis: endocrine regulation of phosphate homeostasis. Nature Reviews Endocrinology.

[145] Reaven G (2002). Metabolic syndrome: pathophysiology and implications for management of cardiovascular disease. Circulation.

[146] Rector F (1983). Sodium, bicarbonate, and chloride absorption by the proximal tubule. American Journal of Physiology - Renal Physiology.

[147] Roberts CK, Hevener AL, Barnard RJ (2013). Metabolic syndrome and insulin resistance: underlying causes and modification by exercise training. Comprehensive Physiology.

[148] Rochlani Y, Pothineni NV, Kovelamudi S, Mehta JL (2017). Metabolic syndrome: pathophysiology, management, and modulation by natural compounds. Therapeutic Advances in Cardiovascular Disease.

[149] Rosenson RS, Brown AS (2002). Statin use in acute coronary syndromes: cellular mechanisms and clinical evidence. Current Opinion in Lipidology.

[150] Ruan X, Zheng F, Guan Y (2008). PPARs and the kidney in metabolic syndrome. American Journal of Physiology - Renal Physiology.

[151] Sanders MJ, Grondin PO, Hegarty BD, Snowden MA, Carling D (2007). Investigating the mechanism for AMP activation of the AMP-activated protein kinase cascade. Biochemical Journal.

[152] Sankri-Tarbichi AG (2012). Obstructive sleep apnea-hypopnea syndrome: Etiology and diagnosis. Avicenna Journal of Medicine.

[153] Sattar N, Lee MM, Kristensen SL, Branch KR, Del Prato S, Khurmi NS (2021). Cardiovascular, mortality, and kidney outcomes with GLP-1 receptor agonists in patients with type 2 diabetes: a systematic review and meta-analysis of randomised trials. The Lancet Diabetes Endocrinology.

[154] Savage DB, Petersen KF, Shulman GI (2007). Disordered lipid metabolism and the pathogenesis of insulin resistance. Physiological Reviews.

[155] Savaş EM, Oğuz SH, Samadi A, Yılmaz Işıkhan S, Ünlütürk U, Lay İ (2020). Apoptosis inhibitor of macrophage, monocyte chemotactic protein-1, and C-reactive protein levels are increased in patients with metabolic syndrome: a pilot study. Metabolic Syndrome and Related Disorders.

[156] Scherer PE, Williams S, Fogliano M, Baldini G, Lodish HF (1995). A novel serum protein similar to C1q, produced exclusively in adipocytes. Journal of Biological Chemistry.

[157] Schlaich M, Straznicky N, Lambert E, Lambert G (2015). Metabolic syndrome: a sympathetic disease?. The Lancet Diabetes Endocrinology.

[158] Schmidt MI, Duncan BB, Sharrett AR, Lindberg G, Savage PJ, Offenbacher S (1999). Markers of inflammation and prediction of diabetes mellitus in adults (Atherosclerosis Risk in Communities study): a cohort study. The Lancet.

[159] Seufert J, Gallwitz B (2014). The extra‐pancreatic effects of GLP‐1 receptor agonists: a focus on the cardiovascular, gastrointestinal and central nervous systems. Diabetes Obesity and Metabolism.

[160] Shahar E, Whitney CW, Redline S, Lee ET, Newman AB, Javier Nieto F (2001). Sleep-disordered breathing and cardiovascular disease: cross-sectional results of the Sleep Heart Health Study. American Journal of Respiratory Critical Care Medicine.

[161] Silveira Rossi JL, Barbalho SM, Reverete de Araujo R, Bechara MD, Sloan KP, Sloan LA (2022). Metabolic syndrome and cardiovascular diseases: Going beyond traditional risk factors. Diabetes/Metabolism Research Reviews.

[162] Singh TG, Samrat Chauhan RC, Singh R, inventor (2023). Deep wound tensile strength measuring apparatus. INDIA2023.

[163] Srivastava RAK, Pinkosky SL, Filippov S, Hanselman JC, Cramer CT, Newton RS (2012). AMP-activated protein kinase: an emerging drug target to regulate imbalances in lipid and carbohydrate metabolism to treat cardio-metabolic diseases: the-matic review series: new lipid and lipoprotein targets for the treatment of cardiometabolic diseases. Journal of Lipid Research.

[164] Stancu C, Sima A (2001). Statins: mechanism of action and effects. Journal of Cellular and Molecular Medicine.

[165] Sugden MC, Bulmer K, Gibbons GF, Holness MJ (2001). Role of peroxisome proliferator-activated receptor-α in the mechanism underlying changes in renal pyruvate dehydrogenase kinase isoform 4 protein expression in starvation and after refeeing. Archives Biochemistry Biophysics.

[166] Sugii S, Evans RM (2011). Epigenetic codes of PPARγ in metabolic disease. FEBS Letters.

[167] Suriyapakorn B, Chairat P, Boonyoprakarn S, Rojanarattanangkul P, Pisetcheep W, Hun-sakunachai N (2019). Comparison of potential drug-drug interactions with metabolic syndrome medica-tions detected by two databases. PloS One.

[168] Sypniewska G (2007). Pro-inflammatory and pro-thrombotic factors and metabolic syndrome. EJIFCC.

[169] Tagliabracci VS, Engel JL, Wiley SE, Xiao J, Gonzalez DJ, Nidumanda Appaiah H (2014). Dynamic regulation of FGF23 by Fam20C phosphorylation, GalNAc-T3 glycosylation, and furin proteolysis. Proceedings of the National Academy of Sciences of the U S A.

[170] Thursby E, Juge N (2017). Introduction to the human gut microbiota. Biochemical Journal.

[171] Tonelli M, Sacks F, Pfeffer M, Gao Z, Curhan G (2005). Relation between serum phosphate level and cardiovascular event rate in people with coronary disease. Circulation.

[172] Tremaroli V, Bäckhed F (2012). Functional interactions between the gut microbiota and host metabolism. Nature.

[173] Turnbaugh PJ, Ley RE, Mahowald MA, Magrini V, Mardis ER, Gordon JI (2006). An obesity-associated gut microbiome with increased capacity for energy harvest. Nature.

[174] Tziomalos K, Athyros VG, Karagiannis A, Mikhailidis DP (2010). Endothelial dysfunction in metabolic syndrome: prevalence, pathogenesis and management. Nutrition Metabolism and Cardiovascular Diseases.

[175] Ugur K, Aydin S (2019). Saliva and blood asprosin hormone concentration associated with obesity. International Journal of Endocrinology.

[176] Uysal KT, Wiesbrock SM, Marino MW, Hotamisligil GS (1997). Protection from obesity-induced insulin resistance in mice lacking TNF-α function. Nature.

[177] Varughese AG, Nimkevych O, Uwaifo GI (2014). Hypercortisolism in obesity-associated hypertension. Current Hypertension Reports.

[178] Verlander JW, Madsen KM, Low PS, Allen DP, Tisher CC (1988). Immunocytochemical localization of band 3 protein in the rat collecting duct. American Journal of Physiology - Renal Physiology.

[179] Vilariño-García T, Polonio-González ML, Pérez-Pérez A, Ribalta J, Arrieta F, Aguilar M (2024). Role of Leptin in Obesity, Cardiovascular Disease, and Type 2 Diabetes. International Journal of Molecular Sciences.

[180] Vilsbøll T, Christensen M, Junker AE, Knop FK, Gluud LL (2012). Effects of glucagon-like peptide-1 receptor agonists on weight loss: systematic review and meta-analyses of randomised controlled trials. BMJ.

[181] Wajchenberg BL (2000). Subcutaneous and visceral adipose tissue: their relation to the metabolic syndrome. Endocrine Reviews.

[182] Wexler AG, Goodman AL (2017). An insider's perspective: Bacteroides as a window into the microbiome. Nature Microbiology.

[183] Wilcox G (2005). Insulin and insulin resistance. Clinical Biochemist Reviews.

[184] Woods SC, D'Alessio DA (2008). Central control of body weight and appetite. Journal of Clinical Endocrinology and Metabolism.

[185] Yamaoka K, Tango T (2012). Effects of lifestyle modification on metabolic syndrome: a systematic review and meta-analysis. BMC Medicine.

[186] Yang JW, Kim HS, Choi YW, Kim YM, Kang KW (2018). Therapeutic application of GPR119 ligands in metabolic disorders. Diabetes Obesity and Metabolism.

[187] Yuan M, Li W, Zhu Y, Yu B, Wu J (2020). Aspro-in: a novel player in metabolic diseases. Frontiers in Endocrinology.

[188] Zeisel SH, Da Costa K-A (2009). Choline: an essential nutrient for public health. Nutrition Reviews.

[189] Zhang BB, Zhou G, Li C (2009). AMPK: an emerging drug target for diabetes and the metabolic syndrome. Cell Metabolism.

[190] Zhao J, Wu Y, Rong X, Zheng C, Guo J (2020). Anti-lipolysis induced by insulin in diverse pathophysiologic conditions of adipose tissue. Diabetes Metabolic Syndrome and Obesity.

[191] Zheng SL, Roddick AJ, Aghar-Jaffar R, Shun-Shin MJ, Francis D, Oliver N (2018). Association between use of sodium-glucose cotransporter 2 inhibitors, glucagon-like peptide 1 agonists, and dipeptidyl peptidase 4 inhibitors with all-cause mortality in patients with type 2 diabetes: a systematic review and meta-analysis. Jama.

